# Supplementary material for: One-step generation of tumor models by base editor multiplexing in adult stem cell-derived organoids
Source: Nat Commun. 2023 Aug 17;14:4998. doi: 10.1038/s41467-023-40701-3 (PMC10435570; doi:10.1038/s41467-023-40701-3)
Supplement: Supplementary file 1 — Supplementary Information [file 41467_2023_40701_MOESM1_ESM.pdf]

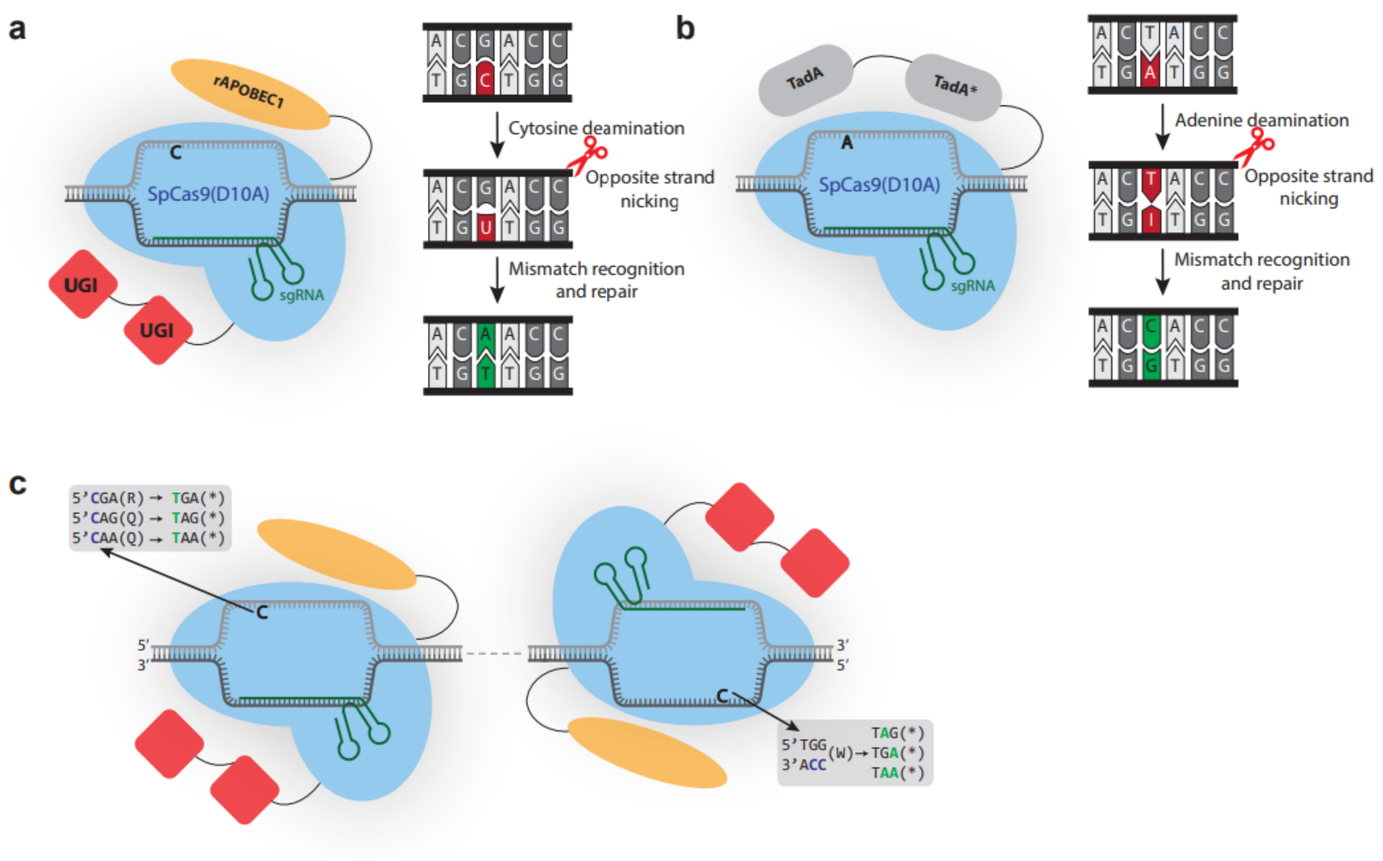

Supplementary figure 1. Principles of Cytosine base editing, Adenine base editing and CRISPR-STOP adapted from [28],[29] and [46].

(a) Cytosine base editor consisting of the nickase Cas9 (D10A) fused to the cytidine deaminase rAPOBEC1 and a tandem repeat of Uracyl Glycosylase inhibitors (UGI). Upon target recognition the DNA opens up in an R-loop upon which the rAPOBEC deaminates Cytosines turning them into Uracil. Uracil is converted to Thymine by nicking the opposite strand to guide DNA repair towards correct C>T editing [28]. **(b)** Adenine base editor consisting of the nickase Cas9 (D10A) fused to a heterodimer of the adenine deaminase TadA. Upon target recognition the DNA opens up in an R-loop upon which TadA deaminates adenines turning them into Inosine. This Inosine residue is converted to Guanine by nicking the opposite strand to guide DNA repair towards correct A>G editing [29]. **(c)** Schematic representation of the CRISPR-stop technique. Arginine (R) and Glutamine (Q) residues can be converted into STOP codons on the sense DNA strand whereas Tryptophan (W) residues can be converted into STOP codons on the anti-sense strand. This will result in knock-out via nonsense mediated decay.

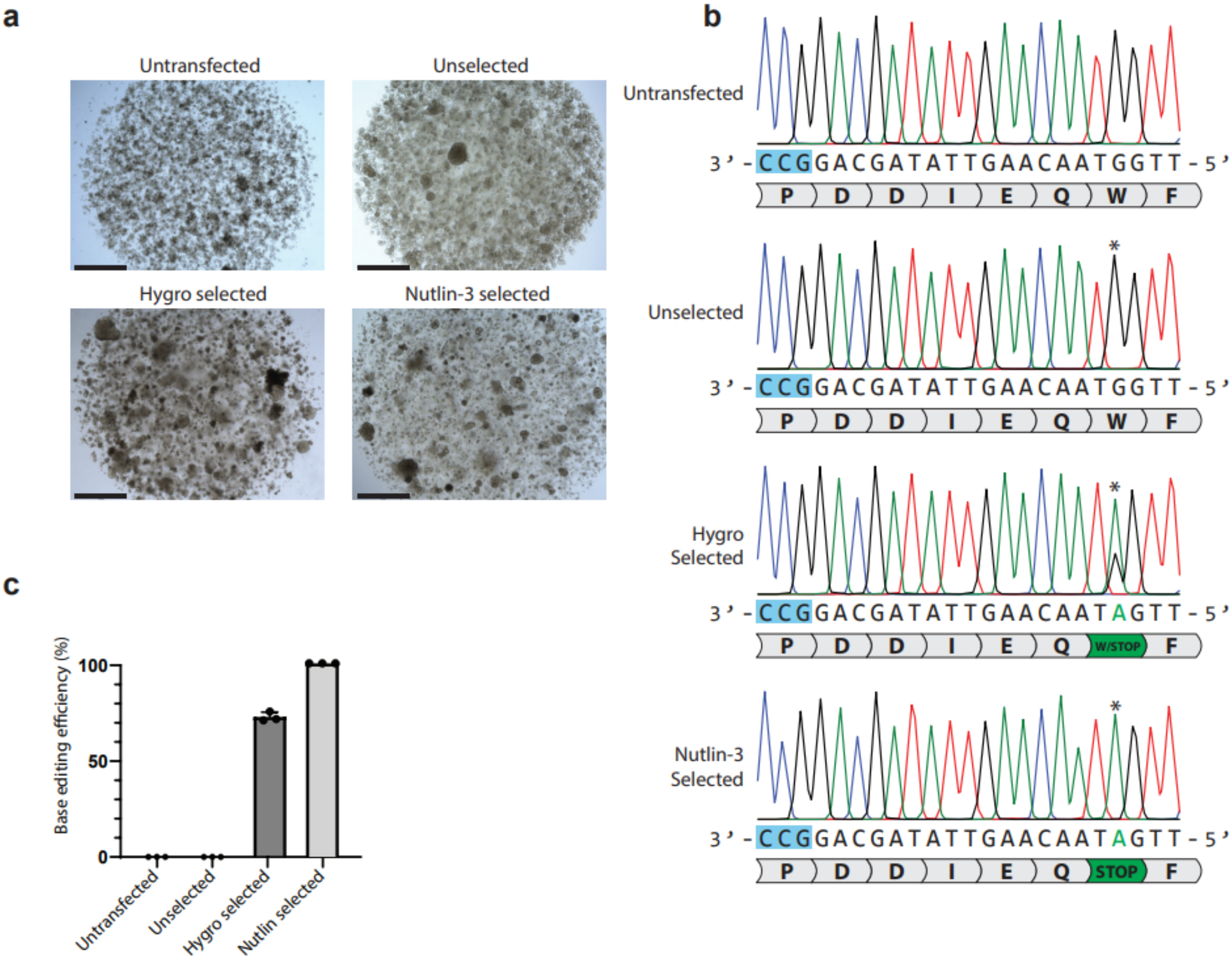

Supplementary figure 2. Hygromycin transposon efficacy testing in fetal hepatocyte organoids.

(a) Brightfield images showing organoids that are untransfected, unselected, hygromycin selected and Nutlin-3 selected. Organoids were transfected with CBE in combination with a *TP53*^{W53*} targeting sgRNA. **(b)** Sanger traces of bulk untransfected, unselected, hygromycin selected and Nutlin-3 selected fetal hepatocyte organoids. **(c)** *In silico* sanger peak quantification by Indigo. n=3 biologically independent samples. Source data are provided as a Source Data file.

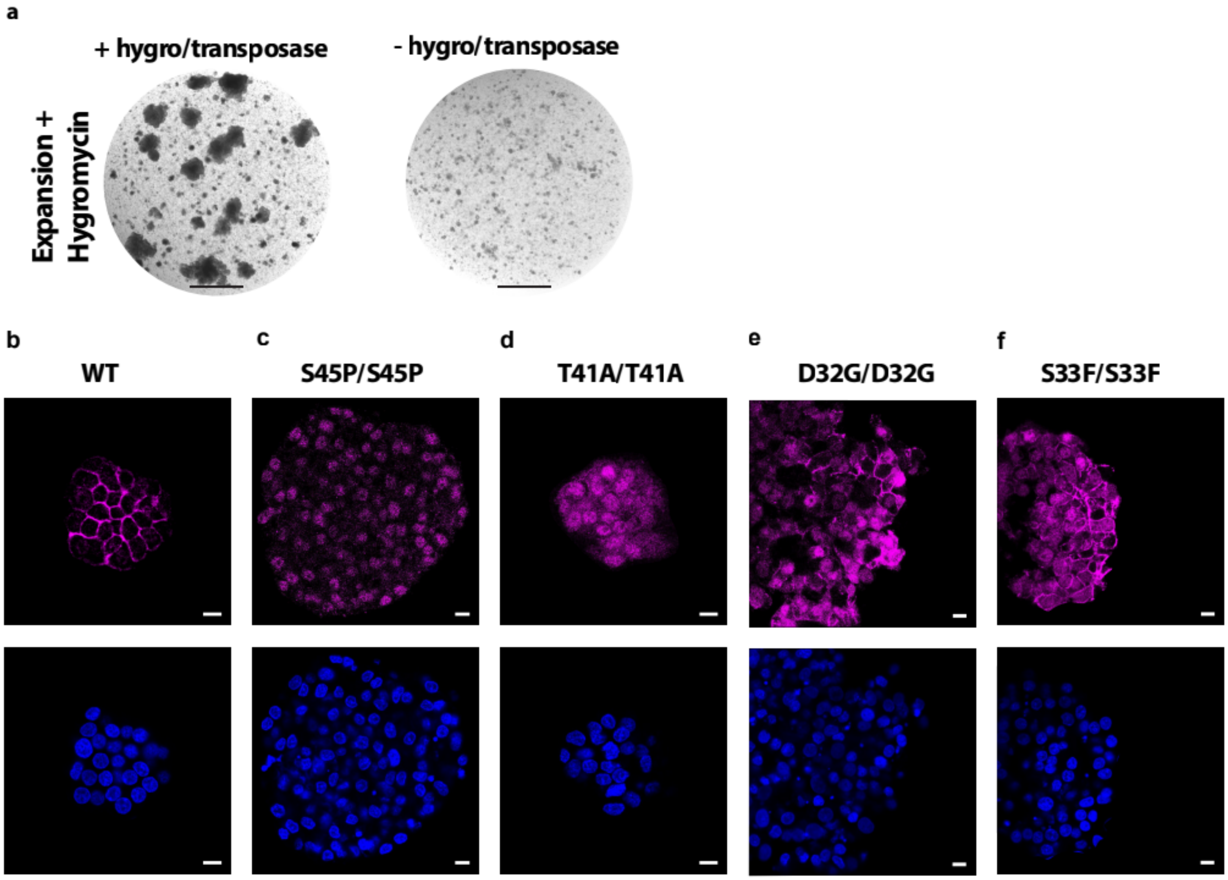

Supplementary figure 3: BE mediated introduction of hot-spot mutations in *CTNNB1* and the impact on localization.
(a) Brightfield images showing clonal hepatocyte organoid outgrowth upon transfection with a plasmid containing hygromycin and transposase. Whereas in control electroporations no organoid clones grow out. **(b)** Confocal microscopy highlighting the localization of CTNNB at the plasma membrane in WT organoids. **(c)** Confocal microscopy highlighting the localization of CTNNB at the plasma membrane in S45P organoids. **(d)** Confocal microscopy highlighting the localization of CTNNB at the plasma membrane in T41A organoids. **(e)** Confocal microscopy highlighting the localization of CTNNB at the plasma membrane in D32G organoids. **(f)** Confocal microscopy highlighting the localization of CTNNB at the plasma membrane in S33F organoids. Scale bars are 50 µm.

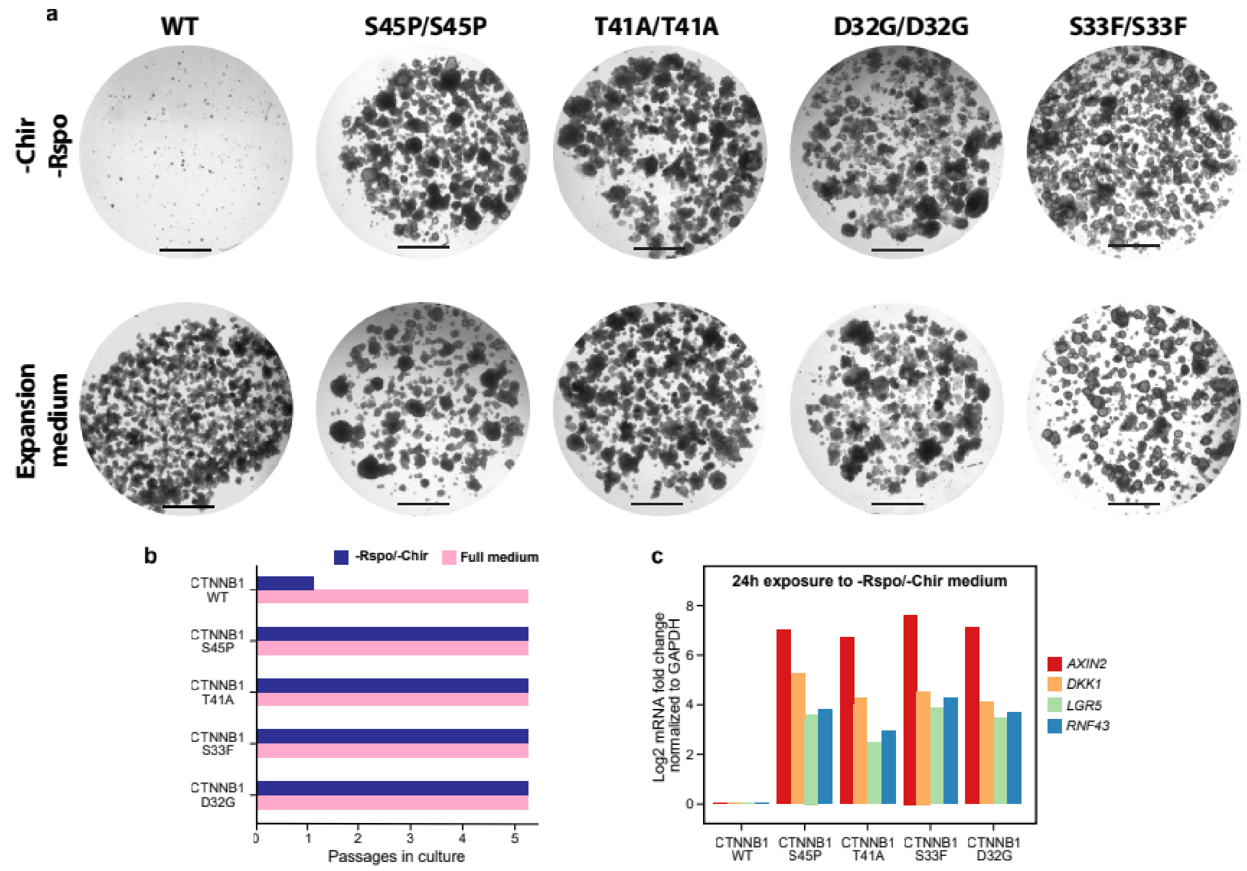

Supplementary figure 4: BE mediated introduction of hot-spot mutations in *CTNNB1*.
(a) Brightfield images showing organoid outgrowth of WT and *CTNBB1* mutant hepatocyte organoids in expansion medium or in medium that lacks Wnt-pathway components CHIR and Rspo-1. **(b)** Quantitative PCR on Wnt-pathway activation genes *AXIN2*, *DKK1*, *LGR5*, and *RNF43* shows several folds increased expression in mutant organoids when cultured in medium without Rspondin-1 and Chir. Expression levels were normalized to *GAPDH* for each condition and log-fold change was calculated relative to the wild type. **(c)** In contrast to wild type organoids, those harboring *CTNNB1* activation mutations can be maintained in culture in medium without Rspondin-1 and Chir. Source data are provided as a Source Data file.

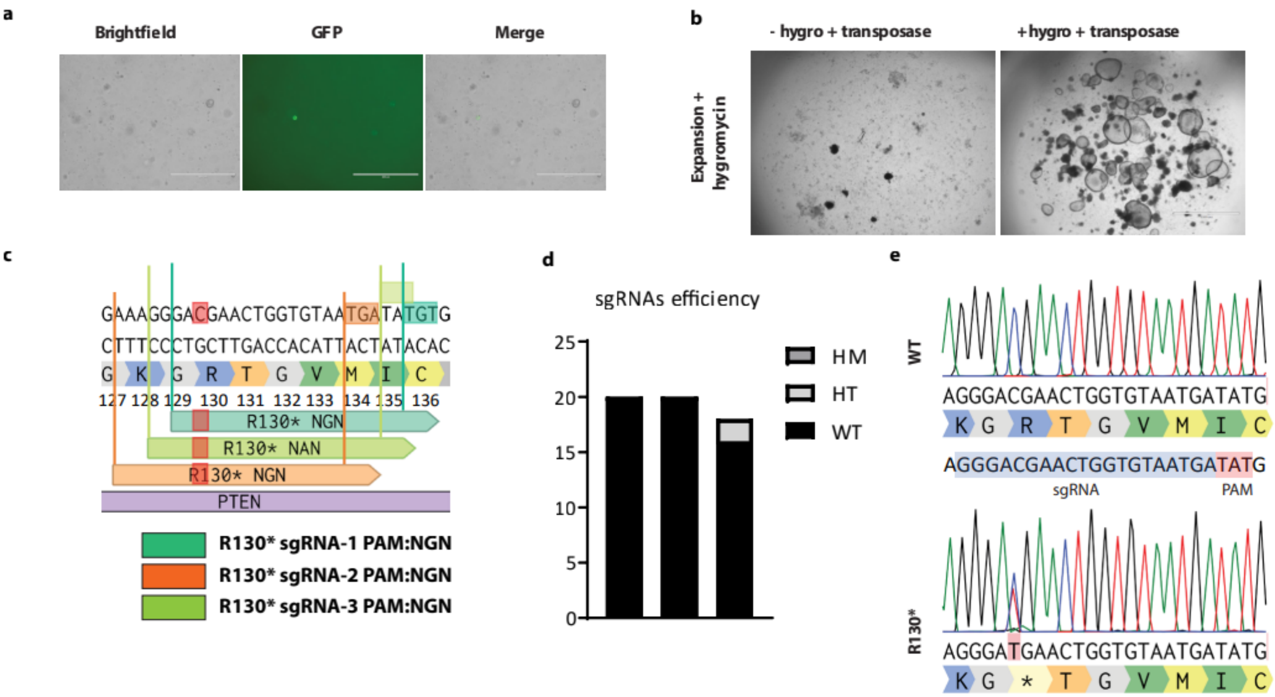

Supplementary figure 5: BE mediated PTEN disruption results in PIK3CA pathway overactivation.
(a) Representative pictures of human endometrial cells electroporated with the CBE carrying a GFP reporter and PTEN sgRNA plasmids. GFP^+ cells were detected at 24h after electroporation. Scale bar 400 µm. **(b)** Representative brightfield pictures of the hygromycin selection of electroporated organoids. Hygromycin resistant clones are visible in presence of a hygromycin resistance cassette. Scale bars are 2000 µm. **(c)** Sequence of the R130 locus in PTEN gene, depicting three possible sgRNA to edit the Cytosine (highlighted in red) in the Arginine (R) codon. The sgRNAs are indicated below the gene sequence while the PAM sequences for each individual guide are highlighted on the gene sequence. Different colors indicate different sgRNA and PAM combinations. **(d)** Bar graph reporting the efficiency of three possible sgRNAs for the *PTEN* R130 locus. Only the SpRY CBE resulted in efficient editing. **(e)** sequence alignment of the *PTEN* R130 locus showing successful C>T transition. The sgRNA sequence is indicated in blue while the PAM sequence is in red. Source data are provided as a Source Data file.

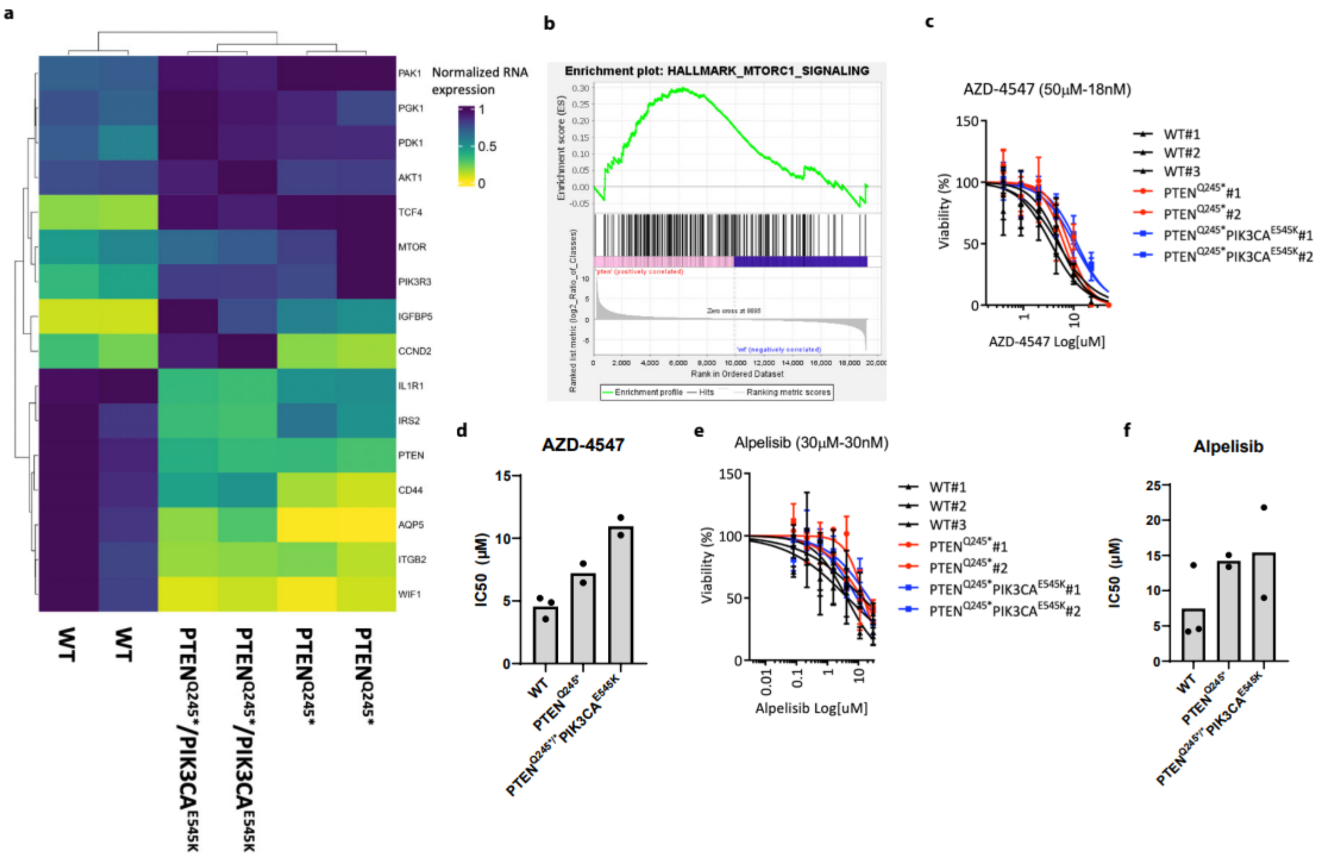

Supplementary figure 6: RNA sequencing and drug sensitivity assays reveal PIK3CA pathway overactivation upon PTEN disruption.

(a) Heatmap showing the selected list of genes extrapolated from the RNA-sequencing analysis on WT and PTEN/PIK3CA mutant organoids, showing altered expression of target genes of the PI3K pathway. The color range represents Normalized RNA expression and ranges from 0 (low) to 1 (high).**(b)** GSE plot generated from the RNA-sequencing analysis describing the upregulation of mTORC1 signaling in mutant organoids. n=3 independent biological samples **(c)** Dose-response curve reporting the sensitivity to AZD-4547 (FGFR inhibitor) of WT and PTEN, PTEN/PIK3CA mutant organoids. The viability is indicated on the Y-axis while the inhibitor concentration is indicated on the X-axis in logarithmic scale. Both mutants show reduced sensitivity. n=3 independent biological samples. "Data are presented as mean vales +/- SD" (**d**) Bar graph representing the IC50 of AZD-4547 in different organoid lines, n=3 (WT), n=2 $PTEN^{Q245*}$ and $PTEN^{Q245*}/PIK3CA^{E545K}$. Statistics derived from three technical replicates per datapoint. **(e)** Dose-response curve reporting the sensitivity to Alpelisib (PIK3CA inhibitor) of WT and PTEN, PTEN/PIK3CA mutant organoids. The viability is indicated on the Y-axis while the inhibitor concentration is indicated on the X-axis in logarithmic scale. Both mutants show reduced sensitivity. n=3 independent biological samples. "Data are presented as mean vales +/- SD" (**f**) Bar graph representing the IC50 of Alpelisib in different organoid lines, n=3 (WT), n=2 PTENQ245* and PTENQ245*/PIK3CAE545K. Statistics derived from three technical replicates per datapoint. Source data are provided as a Source Data file.

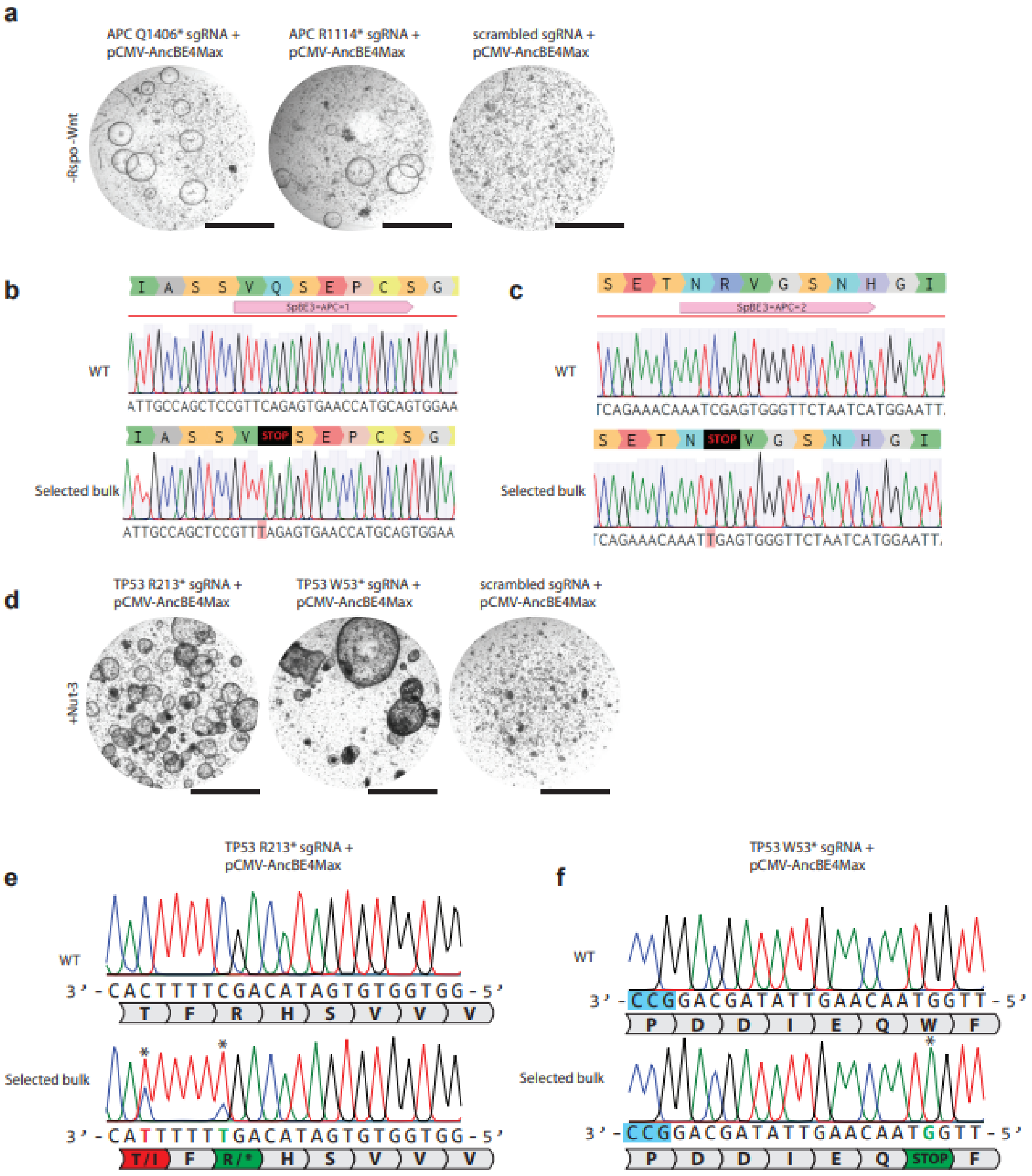

Supplementary figure 7: Optimization of CRISPR-STOP in intestinal organoids by mutation APC and TP53

(a) Brightfield images showing organoid outgrowth upon electroporation of SpCas9-CBE with APC^{Q1406*}, APC^{R1114*} or scrambled control sgRNAs. Scale bars are 2000µm **(b)** Sanger sequencing of bulk organoids upon transfection with APC^{Q1406*} sgRNA. **(c)** Sanger sequencing of bulk organoids upon transfection with APC^{R1114*} sgRNA. **(d)** Brightfield images showing organoid outgrowth upon electroporation of SpCas9-CBE with $TP53^{R213*}$, $TP53\text{-}^{W53*}$ or scrambled control sgRNAs. Scale bars are 2000µm **(e)** Sanger sequencing of bulk organoids upon transfection with $TP53^{R213*}$ sgRNA. The unintended T211I mutation is highlighted in red **(f)** Sanger sequencing of bulk organoids upon transfection with $TP53^{W53*}$ sgRNA. Source data are provided as a Source Data file.

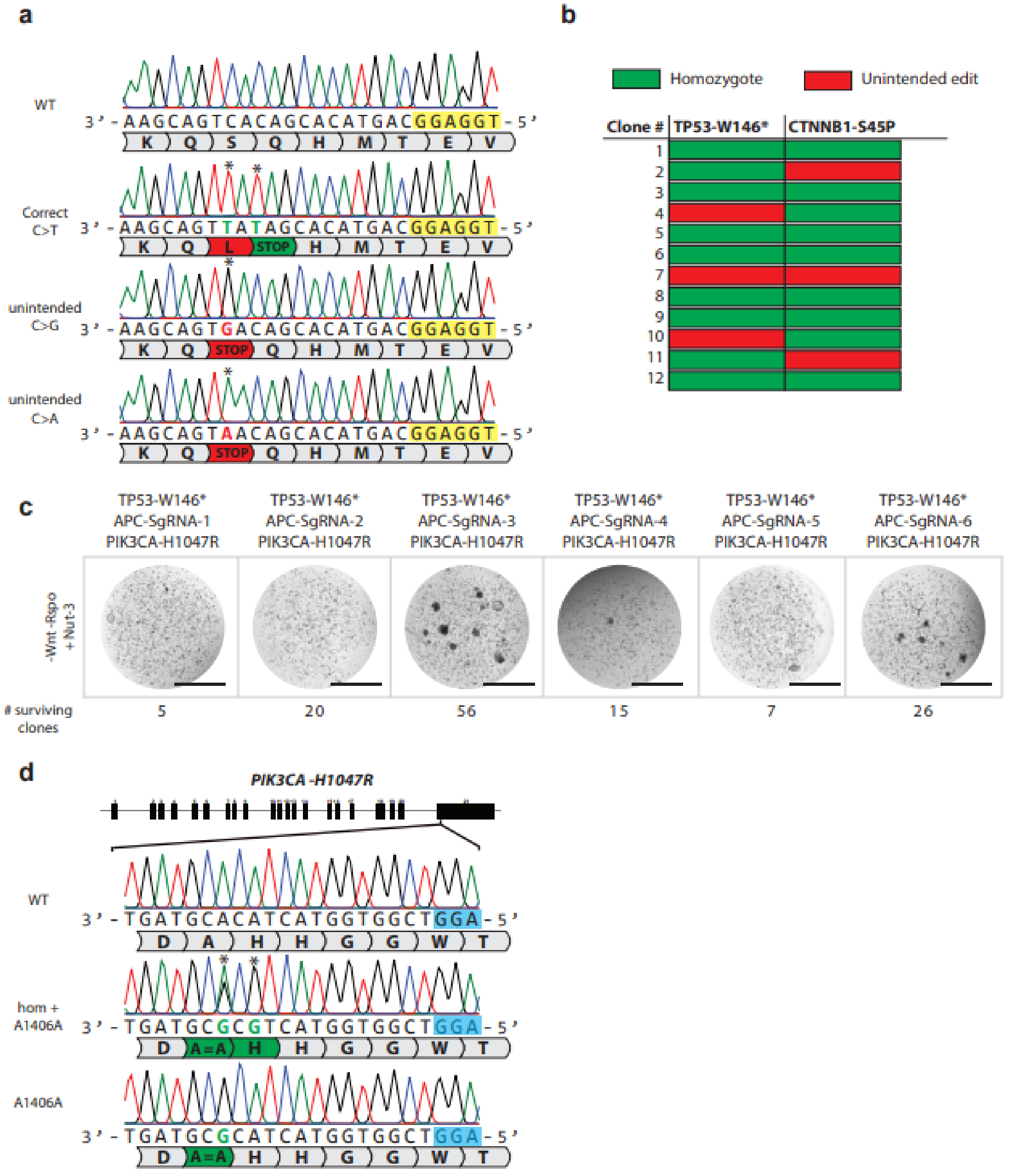

Supplementary figure 8: Optimization of cas9 homolog base editor multiplexing
(a) sanger sequencing of organoids surviving Nutlin-3 selection upon transfection with SaKKH-CBE targeting *TP53*^{Q165*} showing C>T, C>A and C>G editing highlighted in red. **(b)** Sanger sequencing results of 12 individually picked and expanded hepatocyte clones. Green highlights homozygous edit on the locus while red highlights an unintended edit, being non-C>T base editing or indel introduction. **(c)** Brightfield images showing Cas9-homolog multiplexing of SaKKH-CBE targeting *TP53-*^{W146*} and five distinct APC loci. Amount of clones that grew out after selection is shown below. Scale bars are 2000µm **(d)** Sanger sequencing of heterozygous and homozygous *PIK3CA*^{H1047R} mutant organoids upon selection and clonal expansion. Source data are provided as a Source Data file.

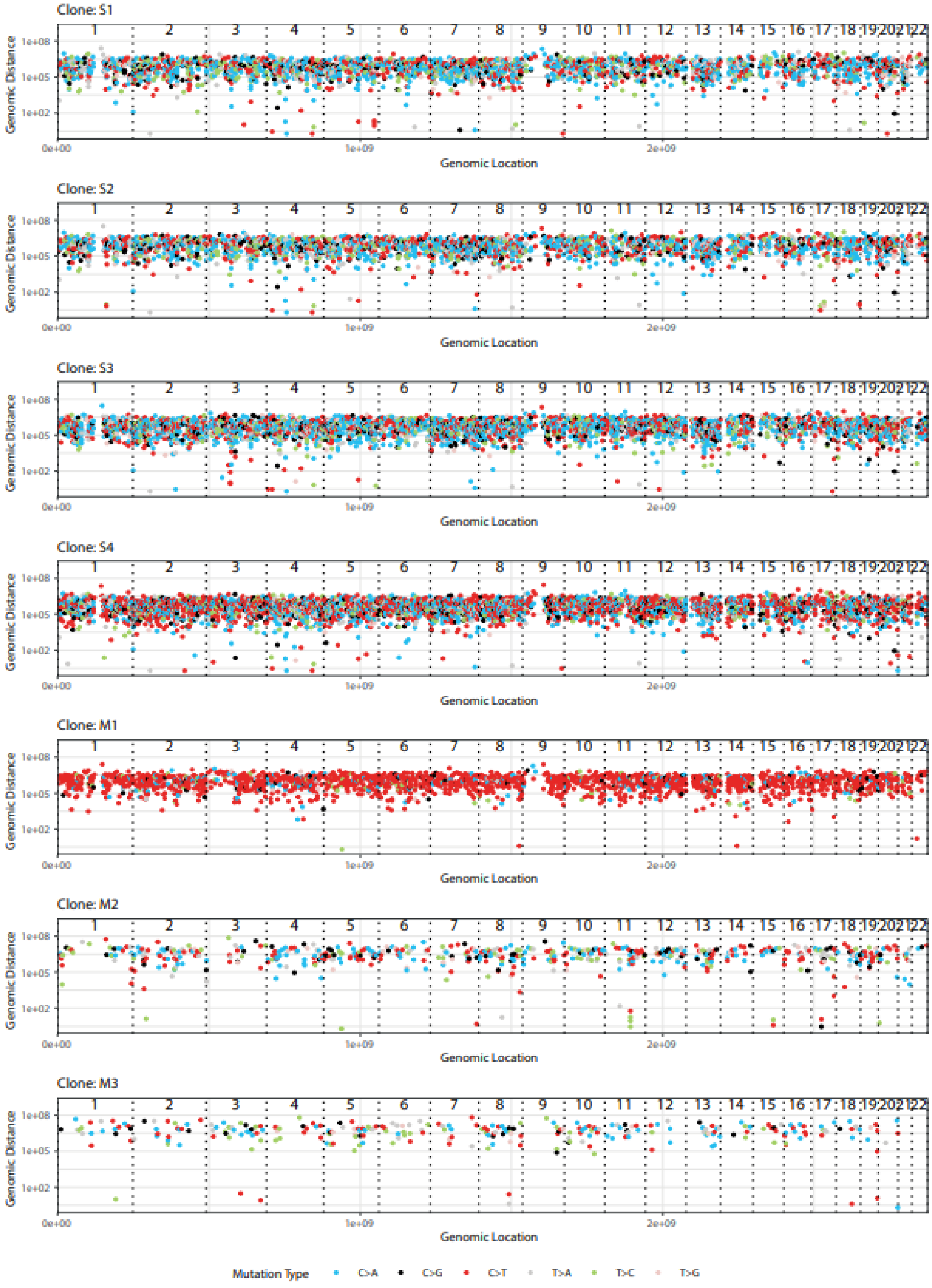

Supplementary figure 9: Rainfall plots of multiplexed and sequentially engineered organoids.

Every identified mutation is indicated with a dot (color according to mutation type) and is ordered on the x-axis from chromosome 1 to chromosome 22. The y-axis shows the distance between each mutation and the one before it (the genomic distance) and is plotted on a log scale

Supplementary Table 1:Genome-wide mutational driver analysis in CBE edited organoids

Clone	#CHROM	POS	ID	REF	ALT	Mutation_type	Gene	nuc_change	aa_change	Effect Prediction	Tumour Types (Somatic) (COSMIC)	ClinVar
S1, S2, S3, S4	7	86864326	.	G	T	missense_variant	GRM3	c.2611G>T	p.Val871Phe	MODERATE	melanoma; oral SCC	Not in ClinVar
S1,	7	148147574	COSM3942108	G	T	missense_variant	CNTNAP2	c.2638G>T	p.Asp880Tyr	MODERATE	glioma; melanoma	Not in ClinVar
S3	14	104773538	.	G	C	missense_variant	AKT1	c.745C>G	p.Arg249Gly	MODERATE	breast; colorectal; ovarian; NSCLC	Not in ClinVar
S3	17	31975543	.	A	G	missense_variant	SUZ12	c.653A>G	p.Asp218Gly	MODERATE	endometrial stromal tumour	Not in ClinVar
S3	17	76736743	.	C	A	sequence_feature	SRSF2	c.326+92G>T		MODERATE	MDS; CLL	Not in ClinVar
S4	1	241517292	.	C	T	missense_variant	FH	c.157G>A	p.Glu53Lys	MODERATE	NA	VCV000214428.7| Uncertain significance (Aug 27, 2021)
S4	19	14099261	.	C	T	sequence_feature	PRKACA	c.420-1371G>A		MODERATE	fibrolamellar hepatocellular carcinoma; cortisol secreting adrenal adenoma	Not in ClinVar

Supplementary Table 2: sgRNA-dependent off-target effects in CBE edited organoids

Mutations in the targeted spacer				
**Clone**	**Zygosity**	**gene**	**Nuc change**	**aa change**
M1,M2,M3	hom	PIK3CA	c.1633G>A	E545K
M1,M2,M3	hom	APC	c.4216C>T	Q1406*
M1,M2,M3	hom (C7), het (C18, C48)	SMAD4	c.1078G>A	D360N
M3	het	SMAD4	c.1082G>A	R361H
S1, S2, S4	hom (CFP2), het (CFP4, CFP11)	FBXW7	c.1435C>T	R479*
S1, S2, S4	hom (CFP2), het (CFP4, CFP11)	FBXW7	c.1433C>T	S478F
S3 and S4	hom (CFP3), het (CFP4)	FBXW7	c.1513C>T	R505C
S3 and S4	hom (CFP3), het (CFP4)	FBXW7	c.1512C>T	V504V
The following mutations are filtered out by GATK 'SnpCluster' Filter, but present in the following clones:				
M3, S1, S2, S4	het	TP53	c.159G>A	W53*
M1, M3, S1, S2, S3, S4	hom	TP53	c.148G>A	W53*
S2, S4	het (CFP4), hom (CFP2)	TP53	c.151G>A	G51K

Mutation in predicted off-target spacer regions (up to 4 mismatches allowed)				
**Clone**				
M1	het	HDAC9	c.25+126487C>T	intronic
M1	het	HDAC9	c.25+126488C>T	intronic
200bp window (targeted)				
**Clone**				
M3	het	PIK3CA	c.1641G>A	E547E
200bp window (off-targets)				
**Clone**				
M1	het	N/A	G>A 5.180391501	N/A

Supplementary Table 3:Primers used for sgRNA construction

Primer name	Primer sequence
universal_SpCas9_FW	/5Phos/GTTTTAGAGCTAGAAATAGCAAGTTAAAATAAGGC
universal_SaCas9_FW	/5Phos/GTTTTAGTACTCTGTAATGAAAATTACAG
CTNNB1_S45P_NGG	CTACCACAGCTCCTTCTCTGcggtgtttcgtcctttccacaag
CTNNB1_T41A_NGA	GAGAAGGAGCTGTGGTAGTGcggtgtttcgtcctttccacaag
CTNNB1_D32S33_NGG	GAATGGATTCCAGAGTCCAGcggtgtttcgtcctttccacaag
PTEN_R130*_TGT	TATCATTACACCAGTTCGTCCcggtgtttcgtcctttccacaag
PTEN_R130*_TGA	TTACACCAGTTCGTCCCTTTCcggtgtttcgtcctttccacaag
PTEN_R130*_TAT	TCATTACACCAGTTCGTCCCCcggtgtttcgtcctttccacaag
PTEN_Q245*_TGG	CACACAGGTAACGGCTGAGGCcggtgtttcgtcctttccacaag
APC_Q1406*_NGG	CTGCATGGTTCACTCTGAACcggtgtttcgtcctttccacaag
APC_R1114*_NGG	TGATTAGAACCCACTCGATTcggtgtttcgtcctttccacaag
TP53_R213*_NGG	CCACACTATGTCGAAAAGTGcggtgtttcgtcctttccacaag
TP53_W53*_NGG	GACGATATTGAACAATGGTTcggtgtttcgtcctttccacaag
PIK3CA_E545K_NGG	CTCTCTGAAATCACTGAGCAcggtgtttcgtcctttccacaag
PIK3CA_H1047R_NGN	AGCCACCATGATGTGCATCAcggtgtttcgtcctttccacaag
SMAD4_R361H_NGG	TTCTGGAGGAGATCGCTTTTcggtgtttcgtcctttccacaag
SaKKH_TP53_1_Q317*	TGGTTTCTTCTTTGGCTGGGcggtgtttcgtcctttccacaag
SaKKH_TP53_2_W146*	CCTGTGCAGCTGTGGGTTGAcggtgtttcgtcctttccacaag
SaKKH_TP53_3_Q165*	GTCATGTGCTGTGACTGCTTcggtgtttcgtcctttccacaag
SaKKH_TP53_4_Q52*	GTCTTCAGTGAACCATTGTTcggtgtttcgtcctttccacaag
SaKKH_TP53_5_Q38*	TCATCCATTGCTTGGGACGGcggtgtttcgtcctttccacaag
SaKKH_APC_1_R1114*	TGATTAGAACCCACTCGATTcggtgtttcgtcctttccacaag
SaKKH_APC_2_Q1406*	CTGCATGGTTCACTCTGAACcggtgtttcgtcctttccacaag
SaKKH_APC_3_Q1127*	TTGACACAAAGACTGGCTTAcggtgtttcgtcctttccacaag

SaKKH_APC_4_R805*	TATCATCATGTCGATTGGTGcggtgtttcgtcctttccacaag
SaKKH_APC_5_Q1291*	TTCCTGTGTCGTCTGATTACcggtgtttcgtcctttccacaag
SaKKH_APC_6_Q1294*	AGAATCTGCTTCCTGTGTCGcggtgtttcgtcctttccacaag
FBXW7_R479*_NGG	AAGAGTGGCATCTCGAGAACcggtgtttcgtcctttccacaag

Supplementary Table 4:Primers used for genotyping by PCR and subsequent sanger sequencing

Locus	FW	Rev	Seq
PTEN R130	TGCTACCAGTCCGTATAGCGT	TTTCCAGGGACTGAGGGTGG	TCTGAGGTTATCTTTTTACCACAGT
PTEN Q245	TCTGCCACTAGAAGTCTAATTTTGG	GCCTTTTCCTTCAAACAGGATTATT	TCTGCCACTAGAAGTCTAATTTTGG
CTNNB1 Exon3	TGATGGAGTTGGACATGGCCAT	GTAGATGGGATCTGCATGCCCT	GTAGATGGGATCTGCATGCCCT
APC Q1406*	TCTTCAGAATCAGCCAGGCACA	CTGGAAGAACCTGGACCCTCTG	AGTGTCACAGCACCCTAGAACC
APC R1114*	AGCAGTTGAACTCTGGAAGGCA	GGCTGATCCACATGACGTTTCT	GGCTGATCCACATGACGTTTCT
TP53 R213*	TGATTGCTCTTAGGTCTGGCCC	ACTGACAACCACCCTTAACCCC	TGGCCATCTACAAGCAGTCACA
TP53 W53*	CACCCATCTACAGTCCCCCTTG	TGACAGGAAGCCAAAGGGTGAA	CTTGGCTGTCCCAGAATGCAAG
PIK3CA_E545K	ATCATCTGTGAATCCAGAGGGGA	AGTGTCTGTGTGGGAGAAACAA	TGCATGCTGTTCAAAAGGTTGACA
SMAD4 R361H	AAACTGTGTTGTGGAGTGCAAG	AAAAACACCGACAATTAAGATGGA	TGGAGTGCAAGTGAAAGCCTTA
TP53 W146 + Q165	CTGAGGTGTAGACGCCAACT	GACAACCACCCTTAACCCCTC	TTAACCCCTCCTCCCAGAGA
PIK3CA_H1047R	CTCCAAACTGACCAAACTGTTCT	CTAATGCTGTTCATGGATTGTGC	TTCCTATGCAATCGGTCTTTGCC

Supplementary Table 5:QPCR primers used in this study

Gene	Fwd	Rev
AKT	TGCTTTCAGGGCTGCTCAAG	ACCTGGTGTCAGTCTCCGAC
AXIN2	AGTGTGAGGTCCACGGAAAC	CTGGTGCAAAGACATAGCCA
DKK1	GGTATTCCAGAAGAACCACCTTG	CTTGGACCAGAAGTGTCTAGCAC
LGR5	TATGCCTTTGGAAACCTCTC	CACCATTCAGAGTCAGTGTT
mTORC1	GCAGATTTGCCAACTATCTTCGG	CAGCGGTAAAAGTGTCCCCTG
PTEN	GCCGTCAAATCCAGAGGCTA	GGATCAGAGTCAGTGGTGTCA
RNF43	GTGTGTGCCATCTGTCTGGA	CGATGGAACTCATGGAGGCA
ZRNF3	GGACCCGAAACCATGCCTC	TCTGCACCCTTCACATACACC
ACTB	CCTCGCCTTTGCCGATCC	GGTGAGGATGCCTCTCTTGC
GAPDH	GTCTCCTCTGACTTCAACAGCG	ACCACCCTGTTGCTGTAGCCAA
