# Supplementary material for: One-step generation of tumor models by base editor multiplexing in adult stem cell-derived organoids
Source: Nat Commun. 2023 Aug 17;14:4998. doi: 10.1038/s41467-023-40701-3 (PMC10435570; doi:10.1038/s41467-023-40701-3)
Supplement: Supplementary file 3 — Description of Additional Supplementary Files [file 41467_2023_40701_MOESM3_ESM.pdf]

Description of Additional Supplementary Files

Title: Supplementary Code 1
Description: The code used to calculate the targeting scope of base editors in tumor modelling.

Title: Supplementary Data 1 - Organoid morphology after quadruple mutation induction
Description: Brightfield images of all handpicked and genotyped organoids. The genotype of these organoids is visualized in figure 6. Scale bars are 500µm.

Title: Supplementary Data 2 - Variants detected in whole genome sequenced multiplexed and sequentially edited clones
Description: VCF files that were used for data analysis.
