# Supplementary material for: One-step generation of tumor models by base editor multiplexing in adult stem cell-derived organoids
Source: Nat Commun. 2023 Aug 17;14:4998. doi: 10.1038/s41467-023-40701-3 (PMC10435570; doi:10.1038/s41467-023-40701-3)
Supplement: Supplementary file 4 — Supplementary Code 1 [file 41467_2023_40701_MOESM4_ESM.docx]

**Supplementary Code 1:**

library(data.table)

library(maftools)

library(readxl)

library(Biostrings)

library(BSgenome)

library(BSgenome.Hsapiens.UCSC.hg38)

setwd("/Users/gijsvanson/OneDrive - Prinses Maxima Centrum/Maarten/")

# general input

clinvar <- readRDS("clinvar_patho_SNP_maf.RDS")

oncogenes <- read.table("cosmic_cancer_gene_census_09052019.txt")

oncogenes <- oncogenes$V1

tumsupgenes <- read_xlsx("tumor suppressor genes.xlsx")

tumsupgenes <- tumsupgenes$GeneSymbol

# question specific input

pam1 <- DNAString("NGG")

pam2 <- DNAString("NGN")

pam3 <- DNAString("NNNRRT")

change1 <- c("C", "T")

change2 <- c("A", "G")

check_for_editable_genes <- function(clinvar,genes, changes, pams){

#make a list of all genes in clinvar to check against the genes of interest.

all_genes <- unique(clinvar@gene.summary$Hugo_Symbol)

message("started filtering your mutations to get pathogenic mutations in your genes of interest.")

# subset the maf-object to represent only the genes of interest.

new_maf <- filterMaf(clinvar, genes = all_genes[-which(all_genes %in% genes)])

message("Done filtering your mutations to get pathogenic mutations in your genes of interest. Now starting making a dataframe to work from.")

# make the table that will be returned in the end, note that editable is set to false by default.

edit_table_oncogene <- data.frame("Gene" = new_maf@data$Hugo_Symbol, "Chrom" = new_maf@data$Chromosome,

"location" = new_maf@data$Start_Position,

"editable" = FALSE,

"ref" = new_maf@data$Reference_Allele, "alt" = new_maf@data$Tumor_Seq_Allele1)

message(paste0("Dataframe ready for use, now starting to check ", length(edit_table_oncogene$Gene), " mutations to see if they are editable"))

# a short bit of code to make sure the progress is shown during calculations.

percentage <- "0%"

total <-length(edit_table_oncogene$Gene)

for(rownumber in seq(length(edit_table_oncogene$Gene))){

x <- rownumber

if (percentage != paste0(round(x/total*100),"%")) {

message(paste0("Checking mutations at ", percentage, "."))

percentage <- paste0(round(x/total*100),"%")

}

# editable will be the value that can change for each row, so by default we start with false.

editable <- FALSE

# get the changes for this specific mutation

ref <- edit_table_oncogene$ref[x]

alt <- edit_table_oncogene$alt[x]

# You can check multiple changes at once.

for(change in changes){

# The option to change the opposing strand is also taken into account.

for (strand in c("+", "-")) {

if (strand == "+") {

change <- change

}

if (strand == "-"){

# to check the opposing strand, the complement change is used.

change <- complement(DNAString(paste0(change, collapse = "")))

}

# if the change fits, continue to see wheiter there is a PAM-site present.

if(all(ref == as.character(change[1]), alt == as.character(change[2]))){

for (pam in pams) {

if (strand == "+") {

# the PAM-site is searched downstream of the mutation, where the editing window is between 12 and 16 bases from the start of the PAM-site.

match <- matchPattern(pam, getSeq(BSgenome.Hsapiens.UCSC.hg38, paste0("chr", edit_table_oncogene$Chrom[x]),

edit_table_oncogene$location[x]+12,

edit_table_oncogene$location[x]+12+4+nchar(pam)), fixed = FALSE)}

if (strand == "-") {

#for the oposing strand, the pam is searched upstream in stead of downstream.

match <- matchPattern(pam, reverseComplement(getSeq(BSgenome.Hsapiens.UCSC.hg38, paste0("chr", edit_table_oncogene$Chrom[x]),

edit_table_oncogene$location[x]-12-4-nchar(pam),

edit_table_oncogene$location[x]-12)), fixed = FALSE)}

# if one or more PAM-sites are found, the matrix of match will be filled and the mutation thus editable.

if(length(match@ranges)>0){editable <- TRUE}

}}}}

edit_table_oncogene$editable[x] <- editable

}

message("Checking mutations done, here comes the output dataframe.")

return(edit_table_oncogene)

}

# perform the calculations for these two lists of genes.

oncogenes_table <- check_for_editable_genes(clinvar, oncogenes,list(change1,change2), list(pam1,pam2,pam3))

tumsupgenes_table <- check_for_editable_genes(clinvar, tumsupgenes, list(change1,change2), list(pam1,pam2,pam3))

# format the results into a new table to make nice figures

percentage_table_figure <- data.frame("group" = c("oncogenes", "oncogenes", "tumor suppressor genes","tumor suppressor genes"),

"editable" = c("YES", "NO", "YES", "NO"),

"value" = c(length(which(oncogenes_table$editable)),length(which(!oncogenes_table$editable)),

length(which(tumsupgenes_table$editable)),length(which(!tumsupgenes_table$editable))))

# make a piechart for the amount of mutations that can be altered using base editors for the two genesets.

ggplot(percentage_table_figure[c(1,2),]) + geom_bar(aes(x = group, y = value, fill = editable), stat = "identity", width = 1, color = "white") + coord_polar("y" , start = 0) + theme_void() +

ggtitle("Portion of pathogenic SNV in oncogenes that can be altered with base editors",

subtitle = paste0("(n=",length(oncogenes_table$editable),")"))+ scale_fill_manual(values = c("orange2", "pink3"))+

geom_text(aes(x = c(1,1), "y" = c(3300,11000), label = c(paste0(round(percentage_table_figure[1,3]/length(oncogenes_table$editable)*100),"%"),

paste0(round(percentage_table_figure[2,3]/length(oncogenes_table$editable)*100),"%"))), color = "white", size=6, position = "identity")

ggplot(percentage_table_figure[c(3,4),]) + geom_bar(aes(x = group, y = value, fill = editable), stat = "identity", width = 1, color = "white") + coord_polar("y" , start = 0) + theme_void() +

ggtitle("Portion of pathogenic SNV in tumor suppressor genes that can be altered with base editors",

subtitle = paste0("(n=",length(tumsupgenes_table$editable),")"))+ scale_fill_manual(values = c("orange2", "pink3"))+

geom_text(aes(x = c(1,1), "y" = c(3000,9800), label = c(paste0(round(percentage_table_figure[3,3]/length(tumsupgenes_table$editable)*100),"%"),

paste0(round(percentage_table_figure[4,3]/length(tumsupgenes_table$editable)*100),"%"))), color = "white", size=6, position = "identity")

percentage_table_figure2 <- data.frame("group" = c("oncogenes", "oncogenes", "tumor suppressor genes","tumor suppressor genes"),

"editable" = c("YES", "NO", "YES", "NO"),

"value" = c(length(unique(oncogenes_table$Gene[which(oncogenes_table$editable)])),

length(unique(oncogenes_table$Gene)) - length(unique(oncogenes_table$Gene[which(oncogenes_table$editable)])),

length(unique(tumsupgenes_table$Gene[which(tumsupgenes_table$editable)])),

length(unique(tumsupgenes_table$Gene)) - length(unique(tumsupgenes_table$Gene[which(tumsupgenes_table$editable)]))))

ggplot(percentage_table_figure2[c(1,2),]) + geom_bar(aes(x = group, y = value, fill = editable), stat = "identity", width = 1, color = "white") + coord_polar("y" , start = 0) + theme_void() +

ggtitle("Portion of oncogenes that can be altered with base editors",

subtitle = paste0("(n=",sum(percentage_table_figure2[c(1,2),3]),")"))+ scale_fill_manual(values = c("orange2", "pink3"))+

geom_text(aes(x = c(1,1), "y" = c(170,350), label = c(paste0(round(percentage_table_figure2[1,3]/sum(percentage_table_figure2[c(1,2),3])*100),"%"),

paste0(round(percentage_table_figure2[2,3]/sum(percentage_table_figure2[c(1,2),3])*100),"%"))), color = "white", size=6, position = "identity")

ggplot(percentage_table_figure2[c(3,4),]) + geom_bar(aes(x = group, y = value, fill = editable), stat = "identity", width = 1, color = "white") + coord_polar("y" , start = 0) + theme_void() +

ggtitle("Portion of tumor suppressor genes that can be altered with base editors",

subtitle = paste0("(n=",sum(percentage_table_figure2[c(3,4),3]),")"))+ scale_fill_manual(values = c("orange2", "pink3"))+

geom_text(aes(x = c(1,1), "y" = c(175,352), label = c(paste0(round(percentage_table_figure2[3,3]/sum(percentage_table_figure2[c(3,4),3])*100),"%"),

paste0(round(percentage_table_figure2[4,3]/sum(percentage_table_figure2[c(3,4),3])*100),"%"))), color = "white", size=6, position = "identity")
