# Supplementary material for: One-step generation of tumor models by base editor multiplexing in adult stem cell-derived organoids
Source: Nat Commun. 2023 Aug 17;14:4998. doi: 10.1038/s41467-023-40701-3 (PMC10435570; doi:10.1038/s41467-023-40701-3)
Supplement: Supplementary file 5 — Supplementary Data 1 [file 41467_2023_40701_MOESM5_ESM.docx]

| 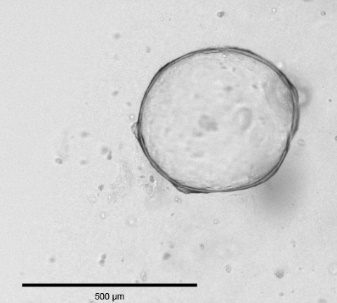**Clone 1** | 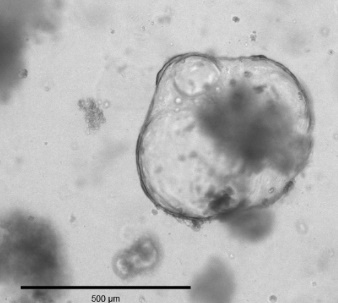**Clone 2** | **Clone 3**  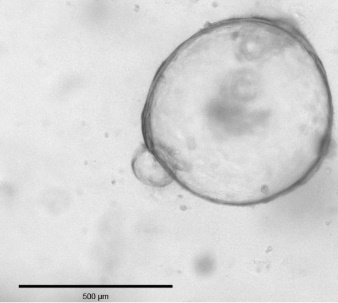 | 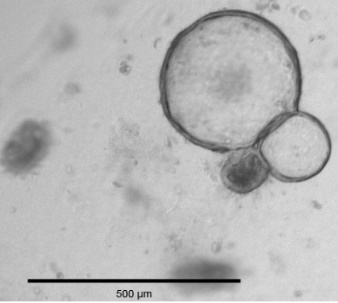**Clone 4** |
| --- | --- | --- | --- |
| 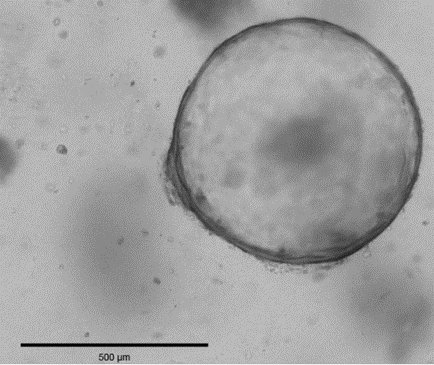**Clone 5** | 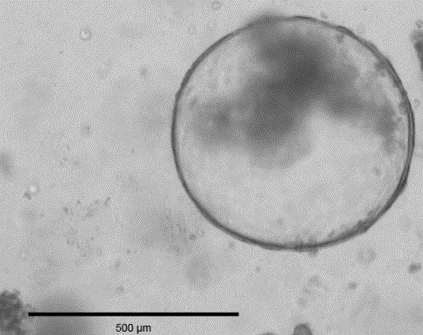**Clone 6** | 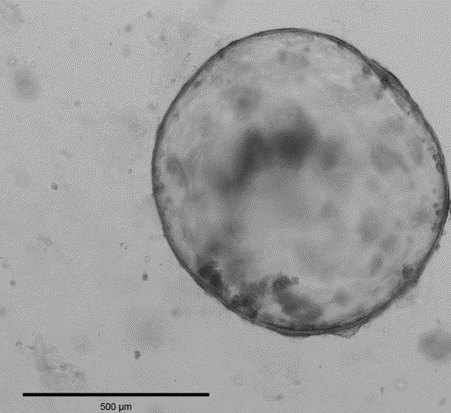**Clone 7** | 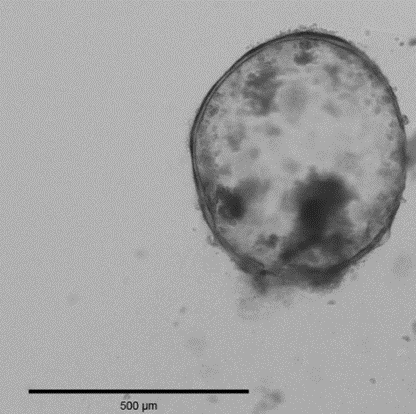**Clone 8** |
| 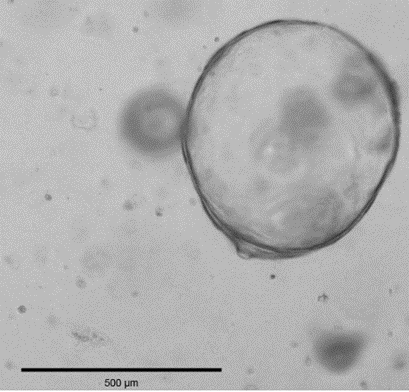**Clone 9** | 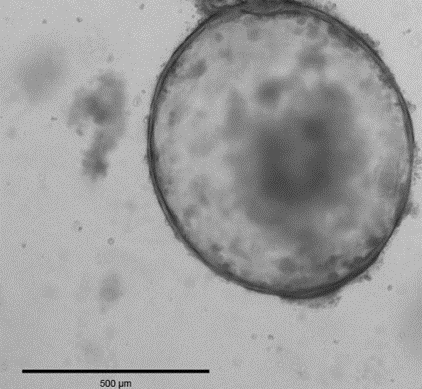**Clone 10** | 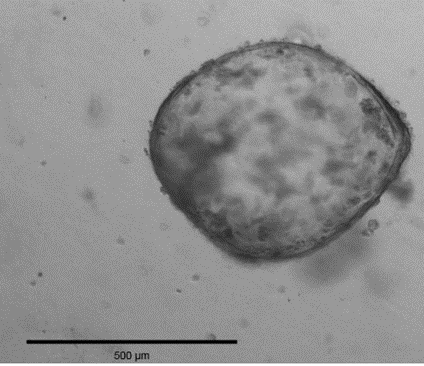**Clone 11** | 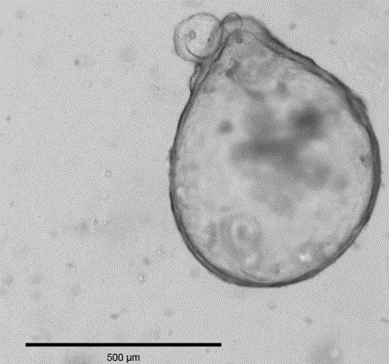**Clone 12** |
| 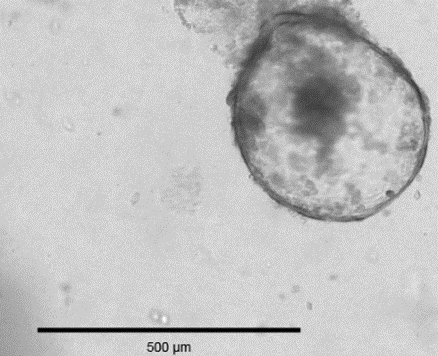**Clone 13** | 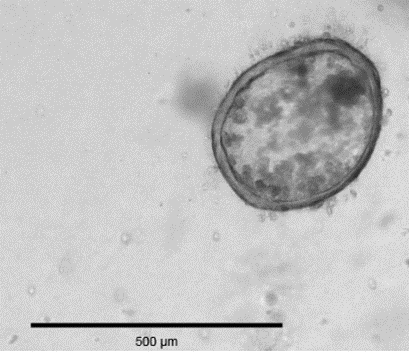**Clone 14** | 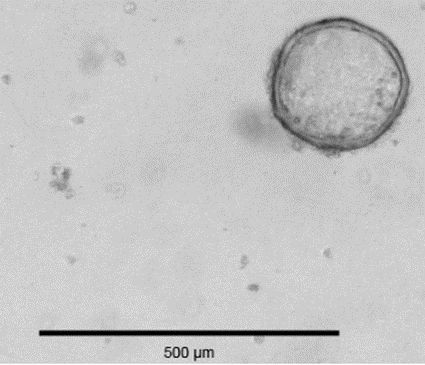**Clone 15** | 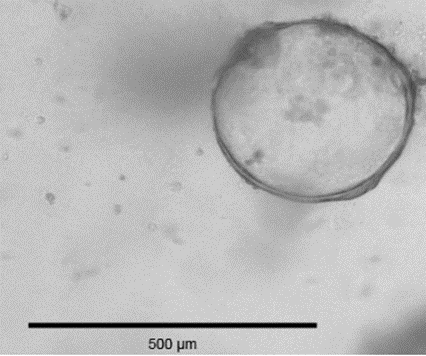**Clone 16** |
| 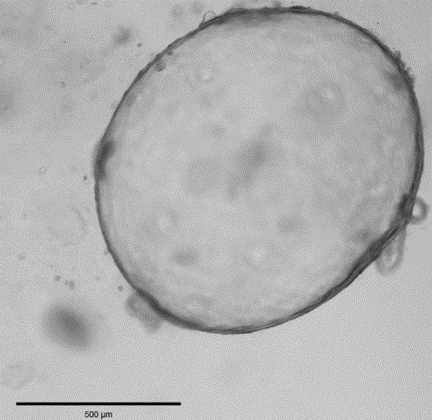**Clone 17** | 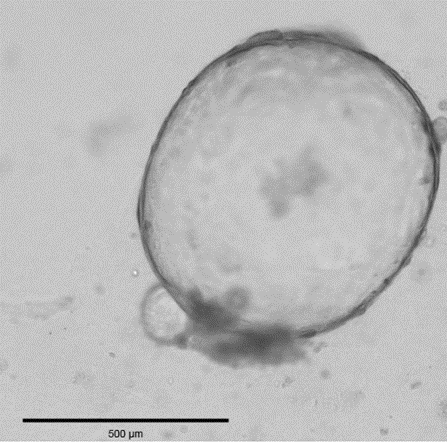**Clone 18** | 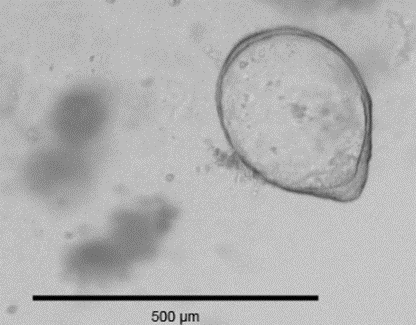**Clone 19** | 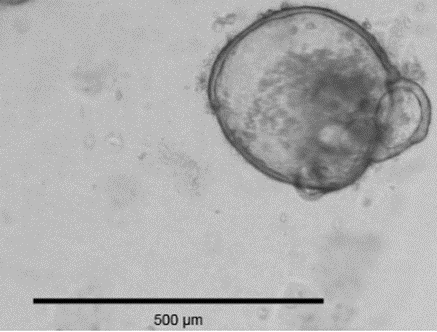**Clone 20** |
| 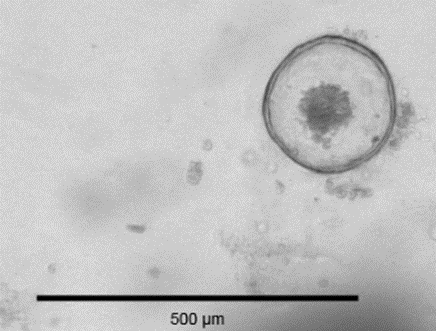**Clone 21** | 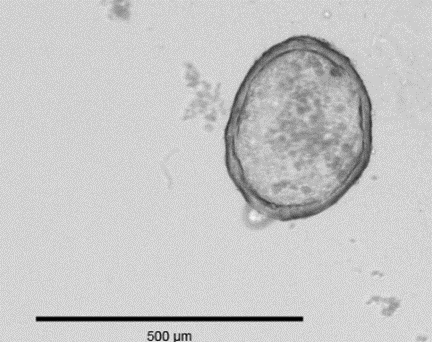**Clone 22** | 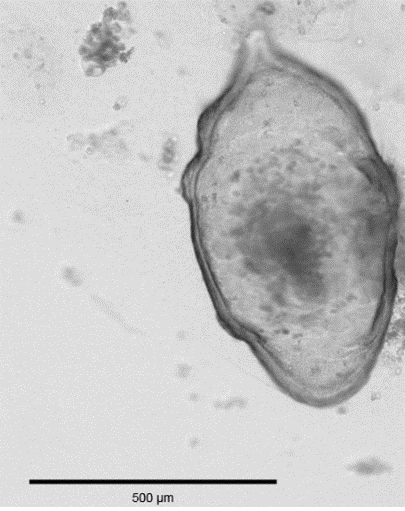**Clone 23** | 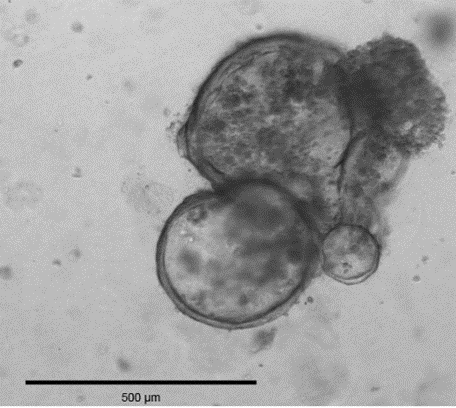**Clone 24** |
| 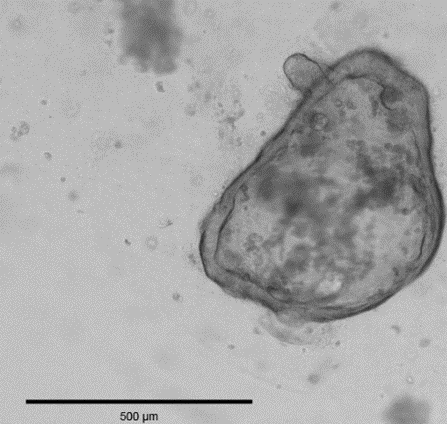**Clone 25** | 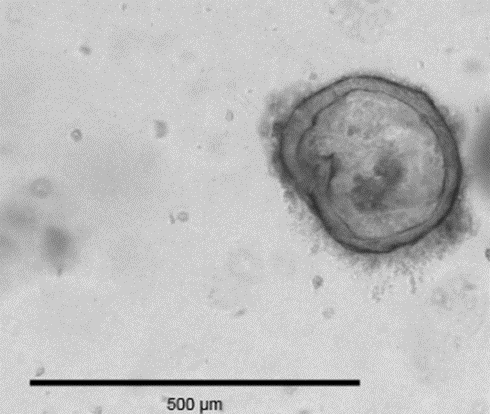**Clone 26** | 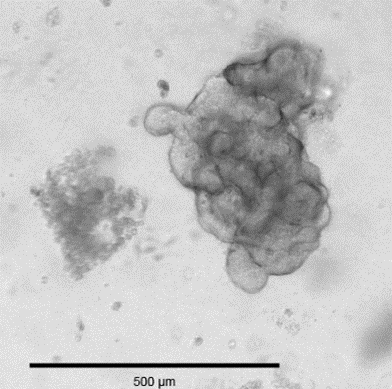**Clone 27** | 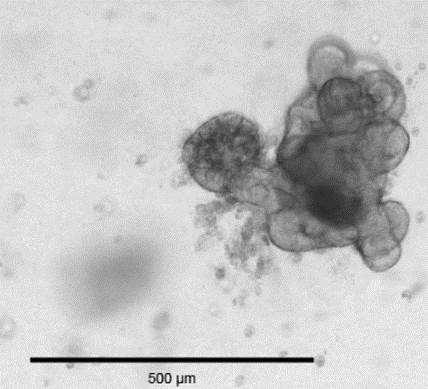**Clone 28** |
| 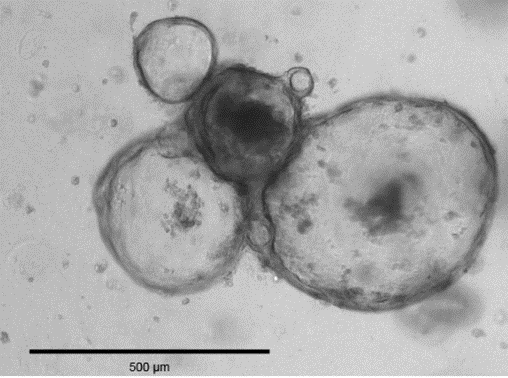**Clone 29** | 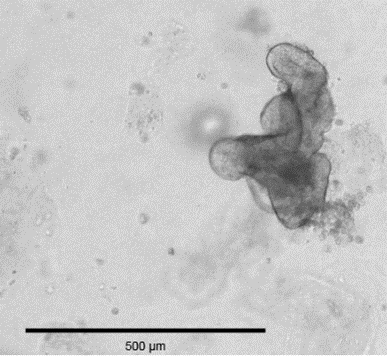**Clone 30** | 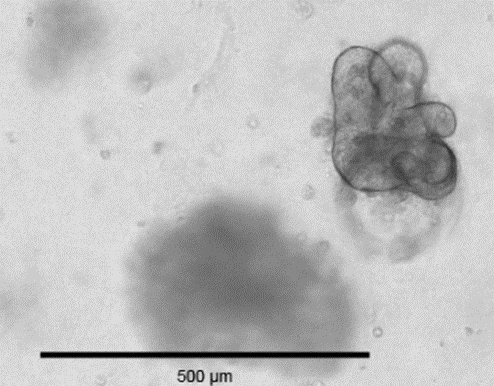**Clone 31** | 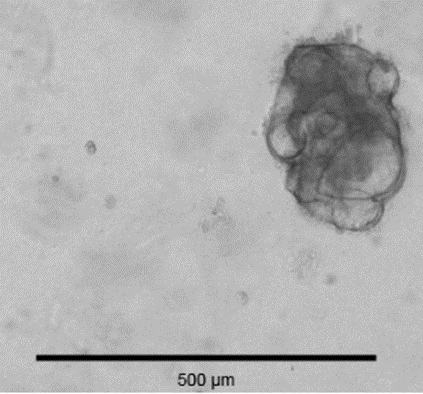**Clone 32** |
| 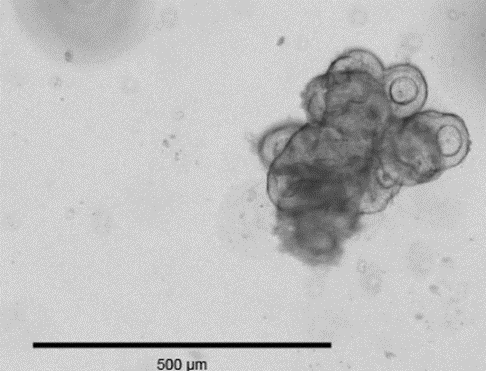**Clone 33** | 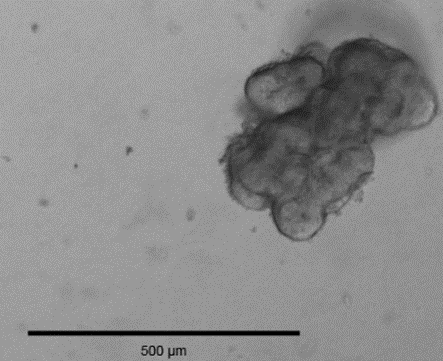**Clone 34** | 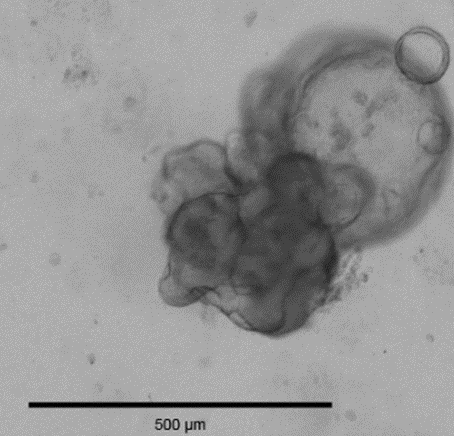**Clone 35** | 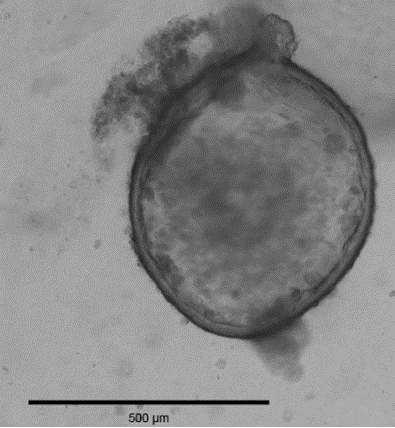**Clone 36** |
| 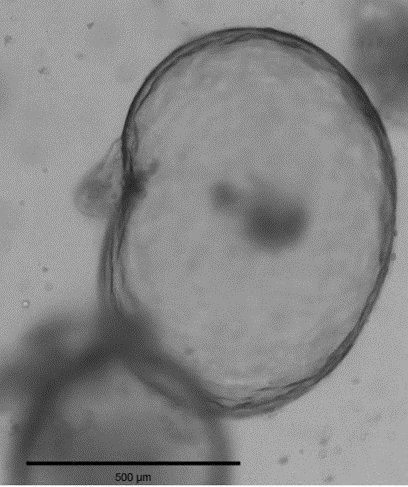**Clone 37** | 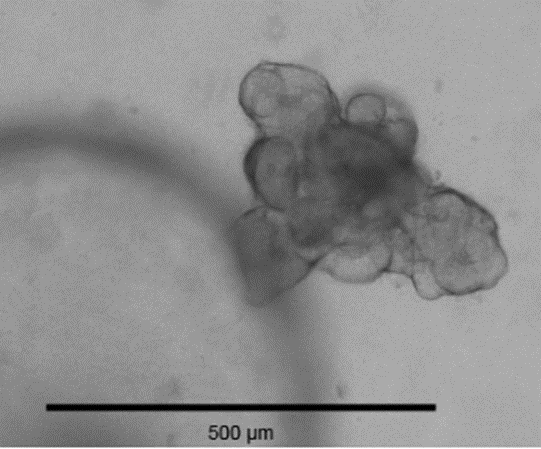**Clone 38** | 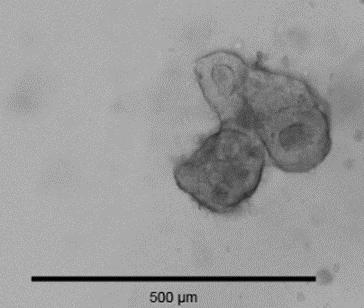**Clone 39** | 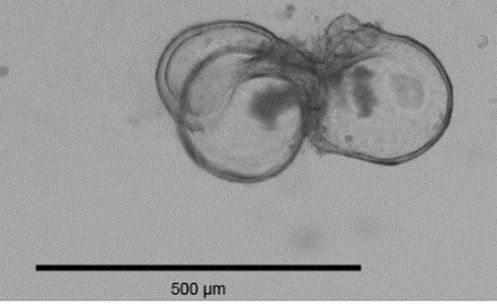**Clone 40** |
| 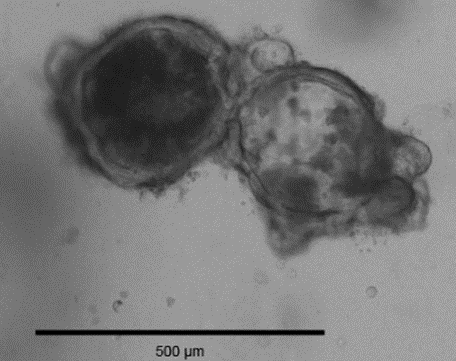**Clone 41** | 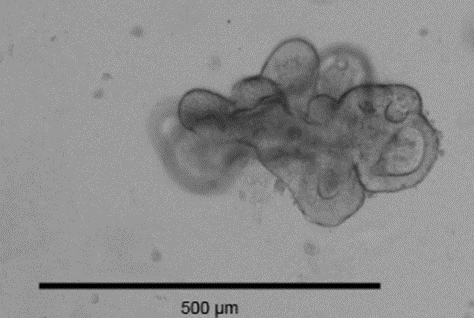**Clone 42** | 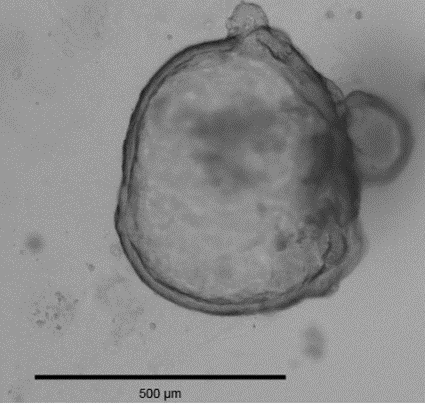**Clone 43** | 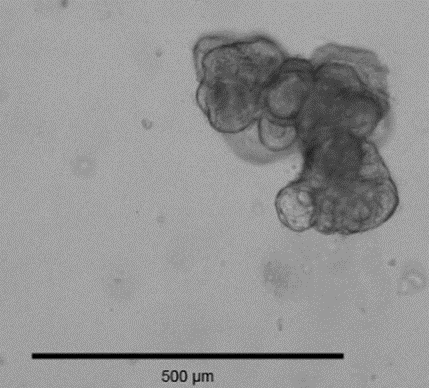**Clone 44** |
| 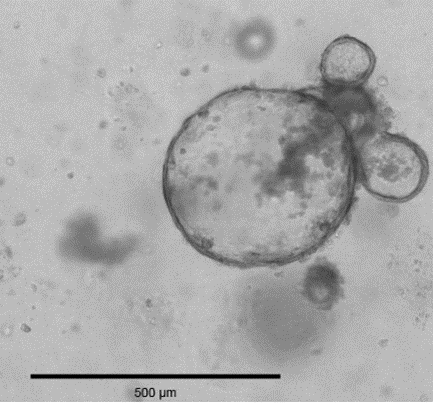**Clone 45** | 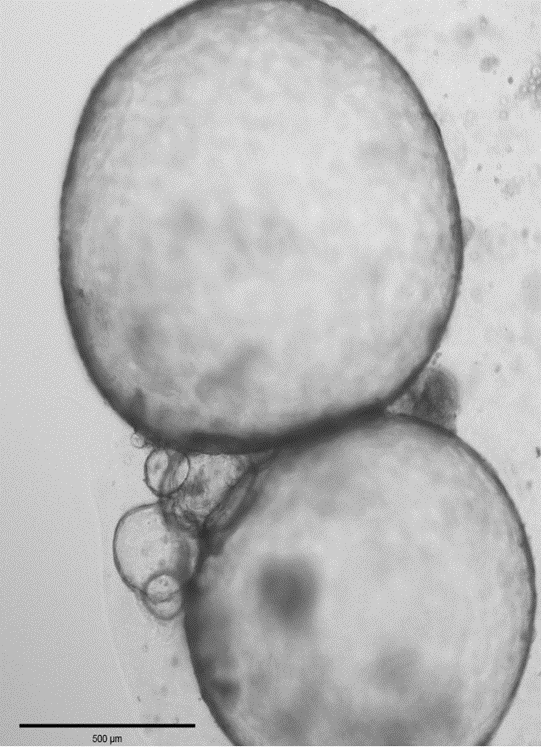**Clone 46** | 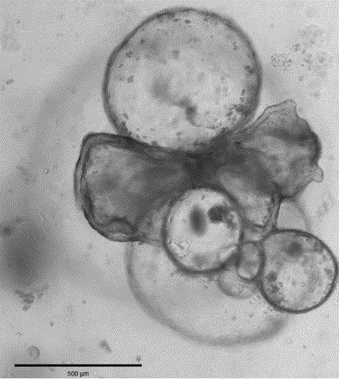**Clone 47** | 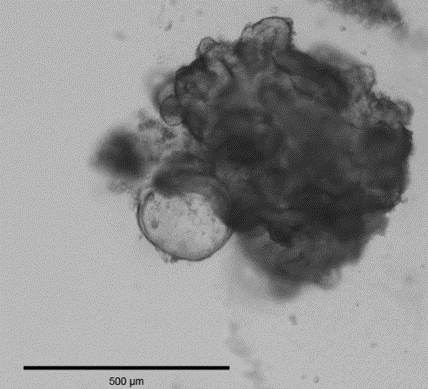**Clone 48** |
| 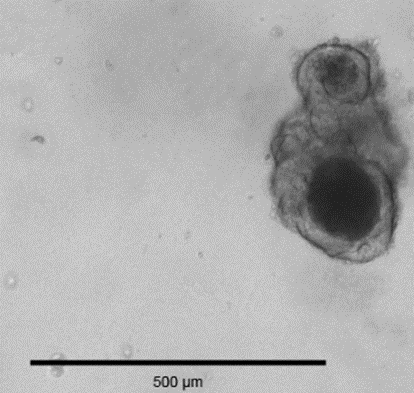**Clone 49** | 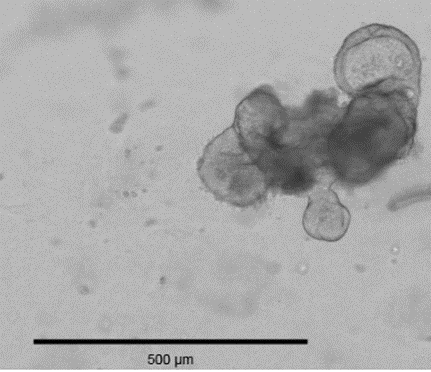**Clone 50** | 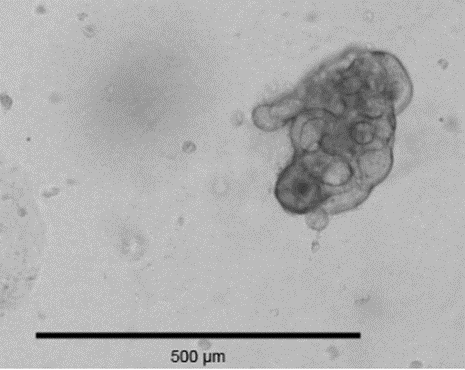**Clone 51** | 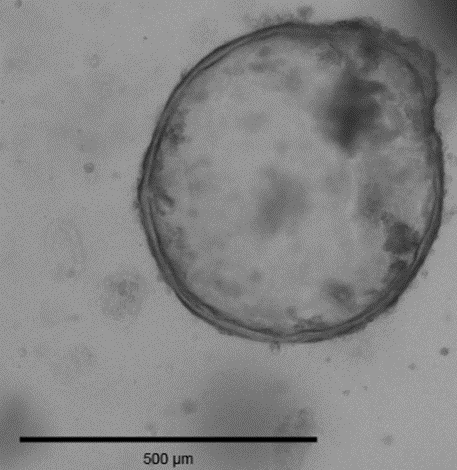**Clone 52** |
| 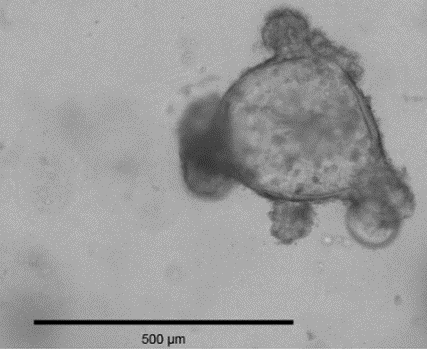**Clone 53** | 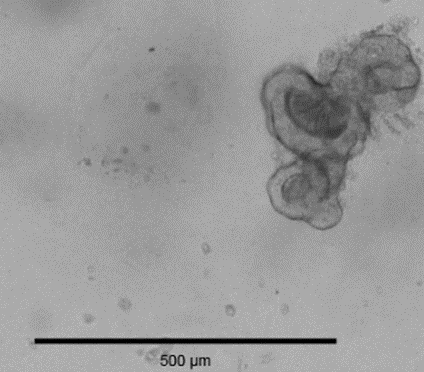**Clone 54** | 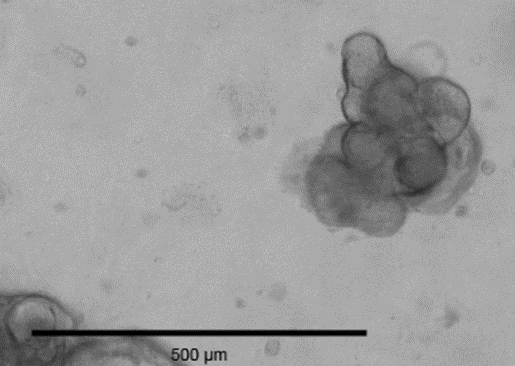**Clone 55** | 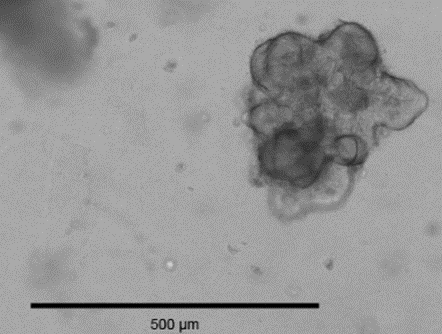**Clone 56** |
| 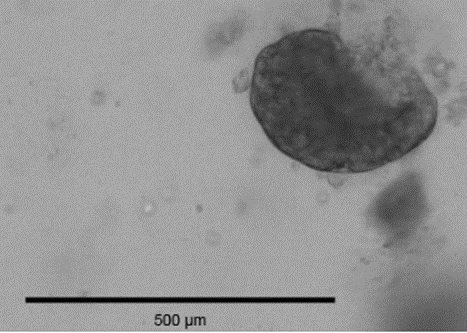**Clone 57** | 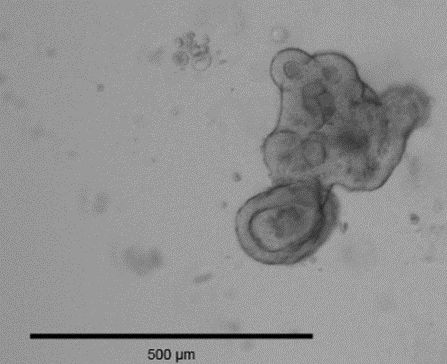**Clone 58** | 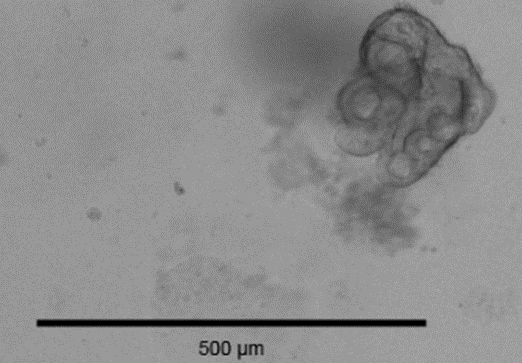**Clone 59** | 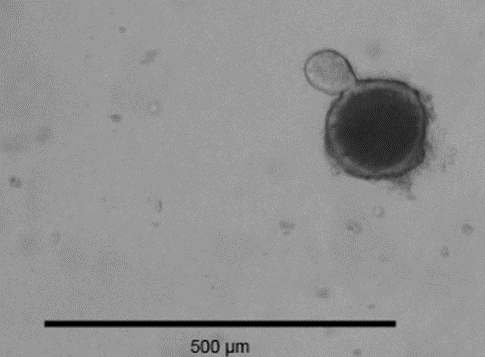**Clone 60** |
| 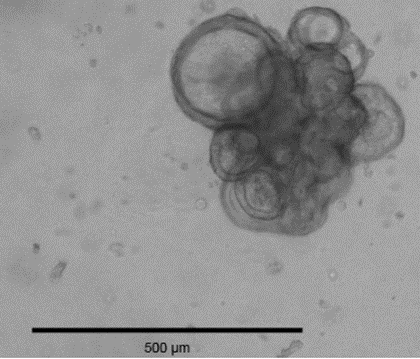**Clone 61** | 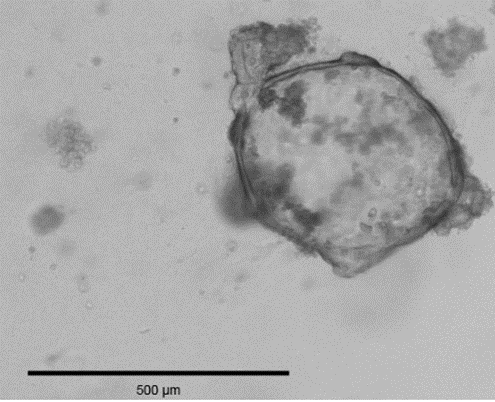**Clone 62** | 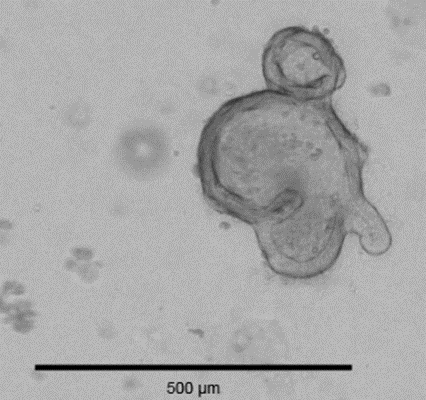**Clone 63** | 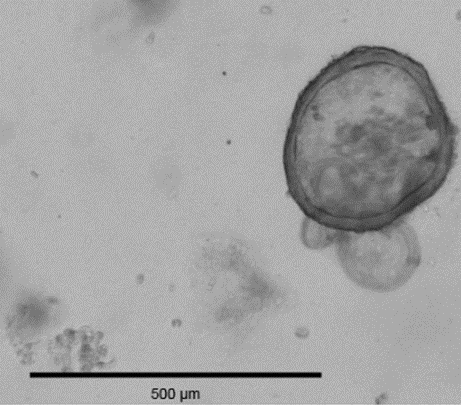**Clone 64** |
| 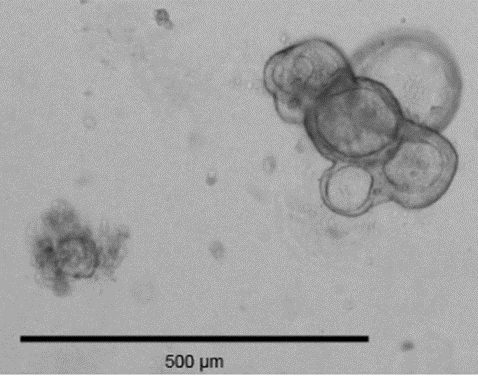**Clone 65** | 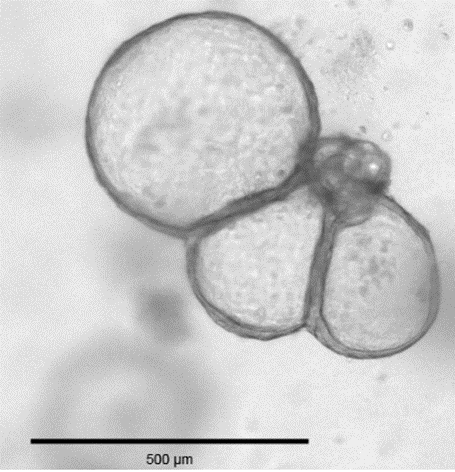**Clone 66** | 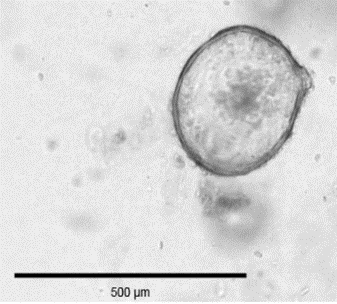**Clone 67** | 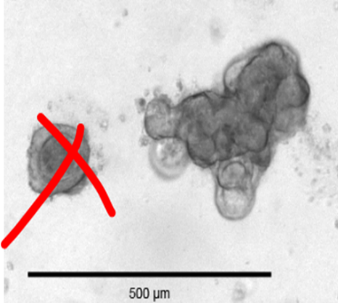**Clone 68** |
| 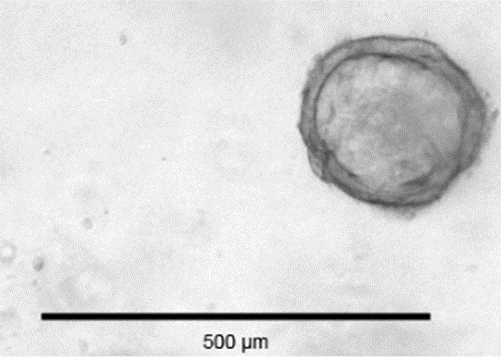**Clone 69** | 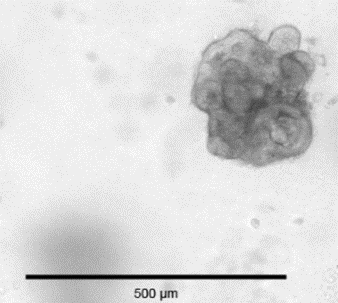**Clone 70** | 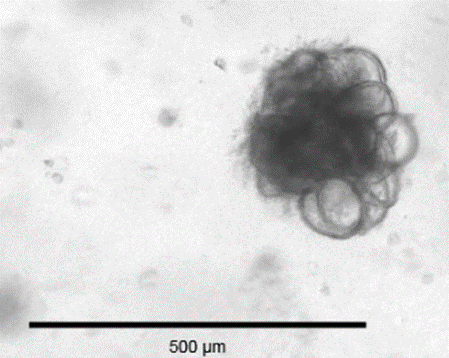**Clone 71** | 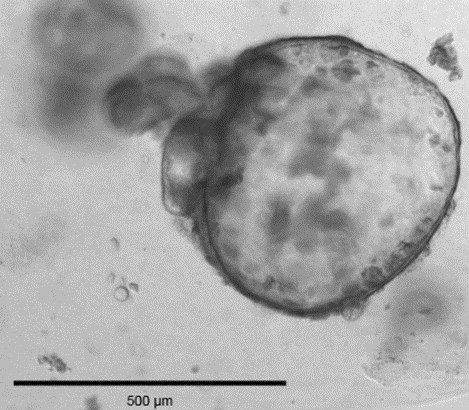**Clone 72** |
| 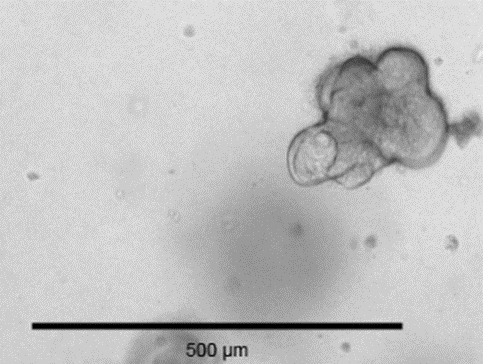**Clone 73** | 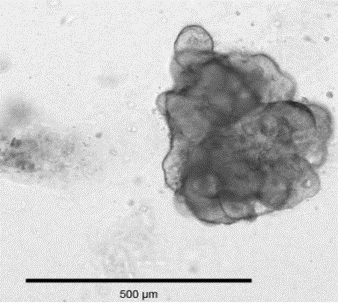**Clone 74** | 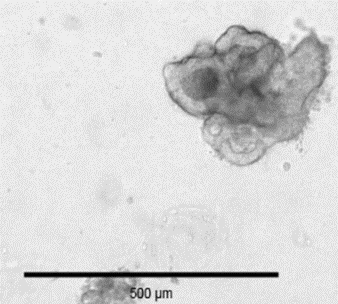**Clone 75** | 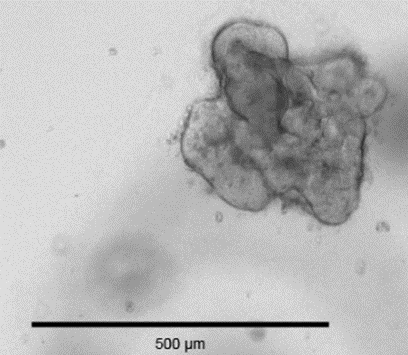**Clone 76** |
| 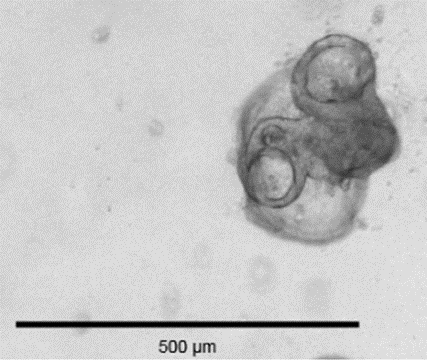**Clone 77** | 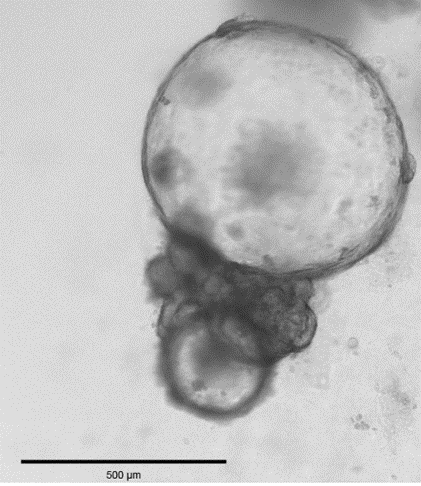**Clone 78** | 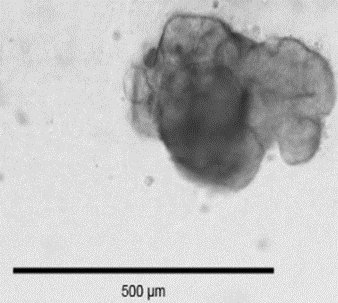**Clone 79** | 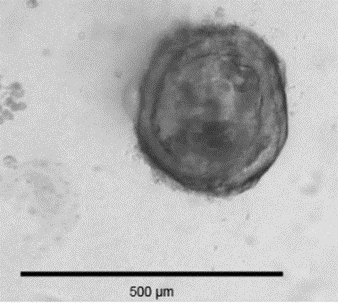**Clone 80** |
| 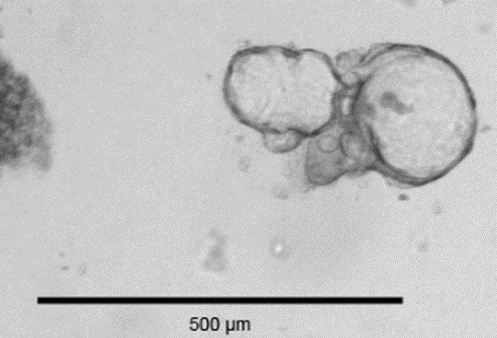**Clone 81** | 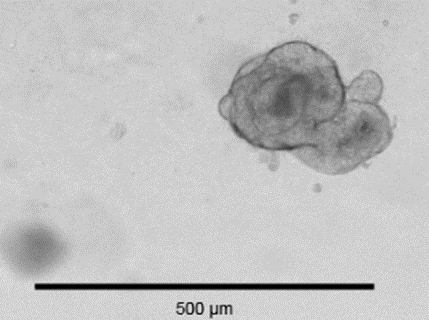**Clone 82** | 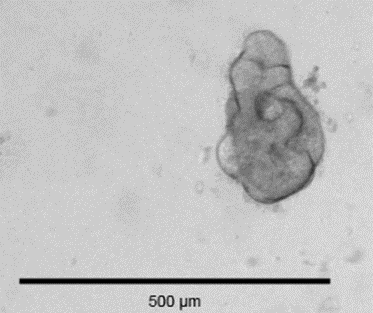**Clone 83** | 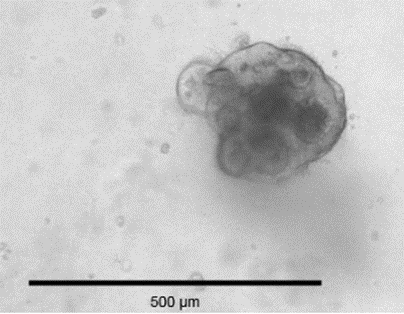**Clone 84** |
| 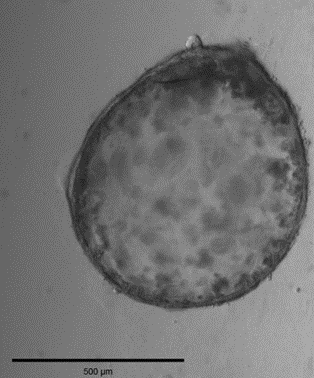**Clone 85** | 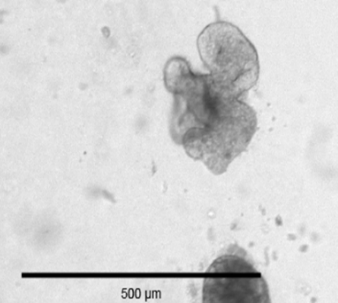**Clone 86** | 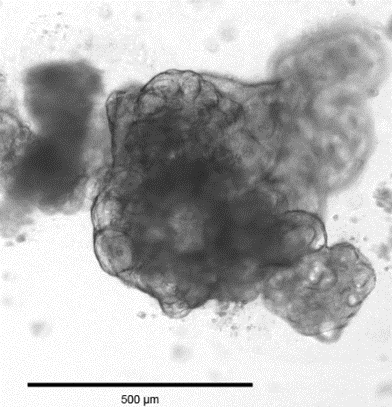**Clone 87** | 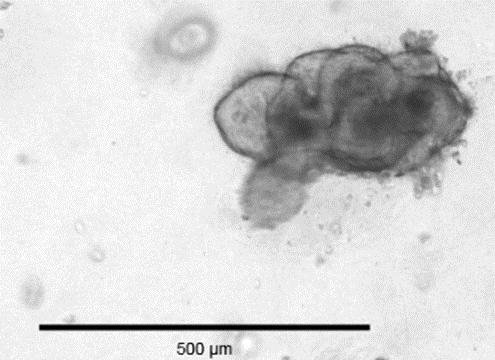**Clone 88** |
| 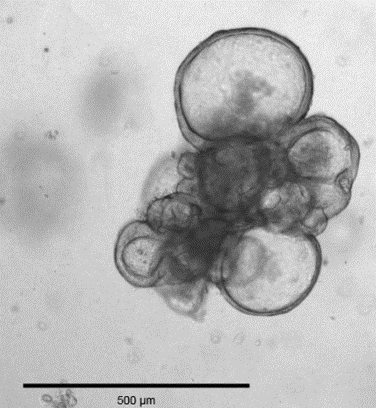**Clone 89** | 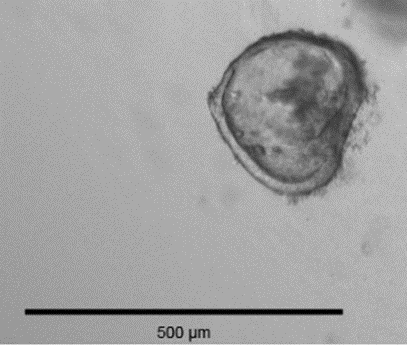**Clone 90** | 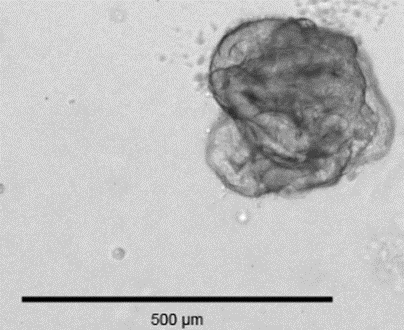**Clone 91** | 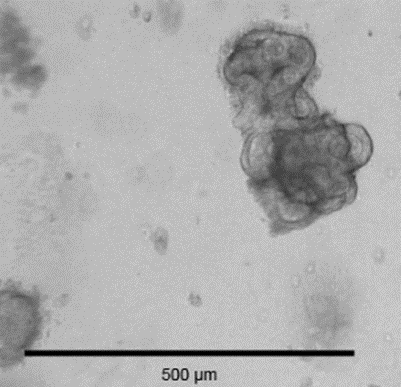**Clone 92** |
| 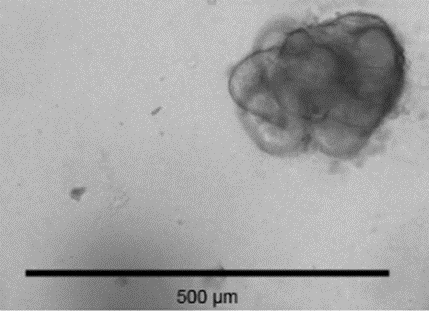**Clone 93** | 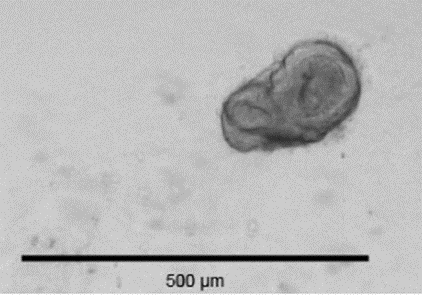**Clone 94** | 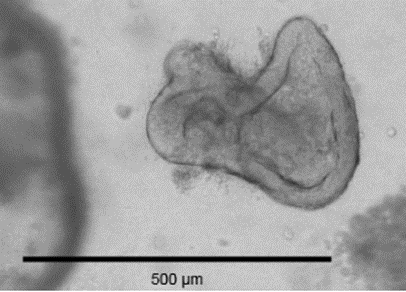**Clone 95** | 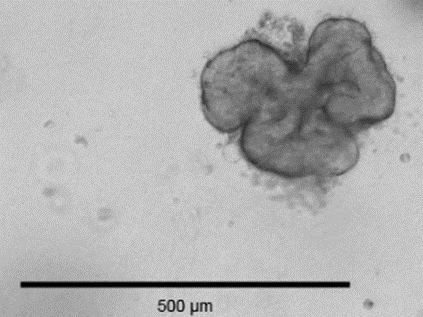**Clone 96** |
