# Supplementary material for: One-step generation of tumor models by base editor multiplexing in adult stem cell-derived organoids
Source: Nat Commun. 2023 Aug 17;14:4998. doi: 10.1038/s41467-023-40701-3 (PMC10435570; doi:10.1038/s41467-023-40701-3)
Supplement: Supplementary file 7 — Reporting Summary [file 41467_2023_40701_MOESM7_ESM.pdf]

nature portfolio

Corresponding author(s): Prof. Dr. Hans Clevers and Dr. Maarten H. Geurts

Last updated by author(s): 06/07/2023

Reporting Summary

Nature Portfolio wishes to improve the reproducibility of the work that we publish. This form provides structure for consistency and transparency in reporting. For further information on Nature Portfolio policies, see our Editorial Policies and the Editorial Policy Checklist.

Statistics

For all statistical analyses, confirm that the following items are present in the figure legend, table legend, main text, or Methods section.

n/a	Confirmed	
☐	☒	The exact sample size (*n*) for each experimental group/condition, given as a discrete number and unit of measurement
☐	☒	A statement on whether measurements were taken from distinct samples or whether the same sample was measured repeatedly
☐	☒	The statistical test(s) used AND whether they are one- or two-sided<br>*Only common tests should be described solely by name; describe more complex techniques in the Methods section.*
☒	☐	A description of all covariates tested
☐	☒	A description of any assumptions or corrections, such as tests of normality and adjustment for multiple comparisons
☐	☒	A full description of the statistical parameters including central tendency (e.g. means) or other basic estimates (e.g. regression coefficient) AND variation (e.g. standard deviation) or associated estimates of uncertainty (e.g. confidence intervals)
☐	☒	For null hypothesis testing, the test statistic (e.g. *F*, *t*, *r*) with confidence intervals, effect sizes, degrees of freedom and *P* value noted<br>*Give P values as exact values whenever suitable.*
☒	☐	For Bayesian analysis, information on the choice of priors and Markov chain Monte Carlo settings
☒	☐	For hierarchical and complex designs, identification of the appropriate level for tests and full reporting of outcomes
☒	☐	Estimates of effect sizes (e.g. Cohen's *d*, Pearson's *r*), indicating how they were calculated

Our web collection on statistics for biologists contains articles on many of the points above.

Software and code

Policy information about availability of computer code

Data collection	The Cancer Gene Census (COSMIC, v2019-05-09) was downloaded from the website (https://cancer.sanger.ac.uk/cosmic/download) directly. The clinvar database (https://www.ncbi.nlm.nih.gov/clinvar/) was downloaded as a RDS file and opened in R(v4.1.3) using read.RDS. Leica LAS X (v1.1) was used for imaging. The Illumina Novaseq 6000 was used for whole genome sequencing.
Data analysis	All analysis, including statistical testing and plotting, was performed in R(v4.1.3) Statistical tests used in the study are mentioned in the main text. Image analysis was performed using FIJI (v1.53)<br><br>For the analysis of whole genome sequencing data all software tools can be found at https://github.com/ToolsVanBox.<br>The following tools were used:<br>Mapping: Burrows-Wheeler Aligner v0.7.17 , duplicate read marking: Sambamba v0.6.8, realignment: Genome Analysis Toolkit (GATK) v4.1.3.0 Variant multisample calling: GATK HaplotypeCaller v4.1.3.0 and GATK-Queue v.4.1.3.0, low-quality and subclonal mutations were excluded by annotating using SMuRF release 2.1.5, Structural variation calling: GRIDSS-purple-linx pipeline v1.3.2, flanking base off-target prediction: BEDtools v2.27.1, Mutational profile detection: Bioconductor R package MutationalPatterns v.3.2.0.

For manuscripts utilizing custom algorithms or software that are central to the research but not yet described in published literature, software must be made available to editors and reviewers. We strongly encourage code deposition in a community repository (e.g. GitHub). See the Nature Portfolio guidelines for submitting code & software for further information.

Data

Policy information about availability of data

All manuscripts must include a data availability statement. This statement should provide the following information, where applicable:

- Accession codes, unique identifiers, or web links for publicly available datasets
- A description of any restrictions on data availability
- For clinical datasets or third party data, please ensure that the statement adheres to our policy

The whole-genome sequencing data from this publication have been deposited to the European Genome-phenome Archive (https://ega-archive.org/) and assigned the identifier: EGAS00001006886.
The RNA sequencing data from this publication have been deposited to the Gene Expression Omnibus, under accession code: GSE236490 (https://www.ncbi.nlm.nih.gov/geo/query/acc.cgi?acc=GSE236490).All software tools used for sequencing data analysis can be found online at: https://github.com/ToolsVanBox.
WGS reads were mapped against the latest version of the human genome GRCh38(https://www.ncbi.nlm.nih.gov/assembly/GCF_000001405.26/)
Variants were filtered for presence in the Cancer Gene Census (COSMIC, v2019-05-09).

Human research participants

Policy information about studies involving human research participants and Sex and Gender in Research.

Reporting on sex and gender	Adult intestinal organoids were derived from a male colorectal cancer patient. At time of operation of the tumor, normal non-transformed mucosa was taken for organoid derivation. The age of the donor for the organoid line used in this study was undisclosed at time of biopsy<br><br>adult endometrium tissue: biopsies were obtained from female patients with different endometrial conditions after informed written consent under the following tissue protocol: 14-472 HUB-Ovarian. The age of the donor of this organoid line was undisclosed at time of biopsy<br><br>hepatocyte tissue: Derived from human fetal liver tissues from the termination material from donors with informed consent under the ethical permission (Leiden University Medical Center)
Population characteristics	Age of donated material is undisclosed upon arrival.
Recruitment	Any available donated intestinal, endometrium and hepatocyte tissue was used to derive organoid lines and stored in biobanks in liquid nitrogen. Organoid lines in this study were selected from these biobanks based on the ease of culturing. Growth speed and splitting ratios may vary depending on the donor.
Ethics oversight	Dutch Ethical Medical Councils (UMC Utrecht, Diakonessenhuis, Leiden University MC).

Note that full information on the approval of the study protocol must also be provided in the manuscript.

Field-specific reporting

Please select the one below that is the best fit for your research. If you are not sure, read the appropriate sections before making your selection.

[x] Life sciences   [ ] Behavioural & social sciences   [ ] Ecological, evolutionary & environmental sciences

For a reference copy of the document with all sections, see nature.com/documents/nr-reporting-summary-flat.pdf

Life sciences study design

All studies must disclose on these points even when the disclosure is negative.

Sample size	No Pre-determined sample sizes were chosen in case of editing efficiency experiments. Editing efficiencies were always determined based on the genotype of individual clones of at least n=2 transfection events. Numbers depend on the survivability of the clones after electroporation. For each experiment we grew out at least 16 individual clonal organoid lines (n) to get a sense of editing efficiency by sanger sequencing. in multiplexing experiments we drastically increased the sample size to 96 (n=96) to get a better sense of co-mutation in our dataset. for WGS at least n=3 individual samples were used for off-target analysis.
Data exclusions	we did not exclude any data in this study
Replication	To verify reproducibility of our strategy we use organoids derived from three distinct human tissues. Editing efficiencies were always

	determined based on the genotype of individual clones of at least n=2 transfection events. sgRNA's were tested prior to multiplexing experiments and effectivity was always reproducible.
Randomization	All of our studies are based on the genotype-phenotype relationships. As the mutated organoids in our study look vastly different compared to wild types, randomization is not sensible.
Blinding	All of our studies are based on the genotype-phenotype relationships. As the mutated organoids in our study look vastly different compared to wild types, blinding is not sensible.

Reporting for specific materials, systems and methods

We require information from authors about some types of materials, experimental systems and methods used in many studies. Here, indicate whether each material, system or method listed is relevant to your study. If you are not sure if a list item applies to your research, read the appropriate section before selecting a response.

Materials & experimental systems

n/a	Involved in the study
☐	☒ Antibodies
☒	☐ Eukaryotic cell lines
☒	☐ Palaeontology and archaeology
☒	☐ Animals and other organisms
☒	☐ Clinical data
☒	☐ Dual use research of concern

Methods

n/a	Involved in the study
☒	☐ ChIP-seq
☒	☐ Flow cytometry
☒	☐ MRI-based neuroimaging

Antibodies

Antibodies used

A) Rabbit anti-CTNNB1, Santa Cruz Biotechnology Cat#Sc-7199; RRID: AB_634603
B) Goat anti-rabbit Alexa Fluor 647, Invitrogen A21245
C) Phalloidin Alexa fluor 488, Life technology, A12379
D) DAPI, invitrogen D1306
E) PTEN Rabbit anti-human PTEN Cell Signaling Technology 9552
F) p-mTOR Mouse anti-human p-mTOR Santa Cruz sc-293133

Validation

antibodies A,B,C and D have been approved by the suppliers for use in immunofluorescence
A) Rabbit anti-CTNNB1, Santa Cruz Biotechnology Cat#Sc-7199; RRID: AB_634603
β-catenin (H-102) is recommended for detection of β-catenin of mouse, rat, human, Xenopus laevis and zebrafish origin by Western Blotting (starting dilution 1:200, dilution range 1:100-1:1000), immunoprecipitation [1-2 µg per 100-500 µg of total protein (1 ml of cell lysate)], immunofluorescence (starting dilution 1:50, dilution range 1:50-1:500), immunohistochemistry (including paraffin-embedded sections) (starting dilution 1:50, dilution range 1:50-1:500) and solid phase ELISA (starting dilution 1:30, dilution range 1:30-1:3000). β-catenin (H-102) is also recommended for detection of β-catenin in additional species, including equine, canine, bovine, porcine and avian.

B) Goat anti-rabbit Alexa Fluor 647, Invitrogen A21245
Anti-Rabbit secondary antibodies are affinity-purified antibodies with well-characterized specificity for rabbit immunoglobulins and are useful in the detection, sorting or purification of its specified target. Secondary antibodies offer increased versatility enabling users to use many detection systems (e.g. HRP, AP, fluorescence). They can also provide greater sensitivity through signal amplification as multiple secondary antibodies can bind to a single primary antibody. Most commonly, secondary antibodies are generated by immunizing the host animal with a pooled population of immunoglobulins from the target species and can be further purified and modified (i.e. immunoaffinity chromatography, antibody fragmentation, label conjugation, etc.) to generate highly specific reagents.

C) Phalloidin Alexa fluor 488, Life technology, A12379
Alexa Fluor 488 phalloidin can be used to visualize and quantitate F-actin in tissue sections, cell cultures, or cell-free preparations. Alexa Fluor 488 phalloidin staining is fully compatible with other fluorescent stains used in cellular analyses, including fluorescent proteins, Qdot nanocrystals and other Alexa Fluor conjugates including secondary antibodies.

D) DAPI, invitrogen D1306
A popular nuclear and chromosome counterstain, DAPI emits blue fluorescence upon binding to AT regions of DNA. Although the dye is cell impermeant, higher concentrations will enter a live cell.

E) PTEN Rabbit anti-human PTEN Cell Signaling Technology 9552
PTEN (138G6) Rabbit mAb detects endogenous levels of total PTEN protein. tested by cell signaling technology for western blot, immunoprecipitation and immunohistochemistry.

F) p-mTOR Mouse anti-human p-mTOR Santa Cruz sc-293133
p-mTOR (59.Ser 2448) is recommended for detection of Ser 2448 phosphorylated mTOR of mouse, rat and human origin by Western Blotting (starting
dilution 1:200, dilution range 1:100-1:1000), immunoprecipitation [1-2 µg
per 100-500 µg of total protein (1 ml of cell lysate)], immunofluorescence
(starting dilution 1:50, dilution range 1:50-1:500), immunohistochemistry
(including paraffin-embedded sections) (starting dilution 1:50, dilution range
1:50-1:500) and solid phase ELISA (starting dilution 1:30, dilution range
1:30-1:3000).
